# Differential effect of cancer-associated fibroblast-derived extracellular vesicles on cisplatin resistance in oral squamous cell carcinoma *via* miR-876-3p

**DOI:** 10.7150/thno.87329

**Published:** 2024-01-01

**Authors:** Soo Hyun Kang, Su Young Oh, Kah-Young Lee, Heon-Jin Lee, Mee-Seon Kim, Tae-Geon Kwon, Jin-Wook Kim, Sung-Tak Lee, So-Young Choi, Su-Hyung Hong

**Affiliations:** 1 Department of Microbiology and Immunology, School of Dentistry, Kyungpook National University, Daegu 700-412, Korea.; 2 Department of Oral Pathology, School of Dentistry, Kyungpook National University, Daegu 700-412, Korea.; 3 Department of Oral and Maxillofacial Surgery, School of Dentistry, Kyungpook National University, Daegu 700-412, Korea.

**Keywords:** cisplatin resistance, cancer-associated fibroblasts, extracellular vesicles, insulin-like growth factor binding protein 3, hsa-miR-876-3p

## Abstract

**Rationale:** Platinum-based chemotherapy is commonly used for treating solid tumors, but drug resistance often limits its effectiveness. Cancer-associated fibroblast (CAF)-derived extracellular vesicle (EV), which carry various miRNAs, have been implicated in chemotherapy resistance. However, the molecular mechanism through which CAFs modulate cisplatin resistance in oral squamous cell carcinoma (OSCC) is not well understood. We employed two distinct primary CAF types with differential impacts on cancer progression: CAF-P, representing a more aggressive cancer-promoting category, and CAF-D, characterized by properties that moderately delay cancer progression. Consequently, we sought to investigate whether the two CAF types differentially affect cisplatin sensitivity and the underlying molecular mechanism.

**Methods:** The secretion profile was examined by utilizing an antibody microarray with conditioned medium obtained from the co-culture of OSCC cells and two types of primary CAFs. The effect of CAF-dependent factors on cisplatin resistance was investigated by utilizing conditioned media (CM) and extracellular vesicle (EVs) derived from CAFs. The impacts of candidate genes were confirmed using gain- and loss-of-function analyses in spheroids and organoids, and a mouse xenograft. Lastly, we compared the expression pattern of the candidate genes in tissues from OSCC patients exhibiting different responses to cisplatin.

**Results:** When OSCC cells were cultured with conditioned media (CM) from the two different CAF groups, cisplatin resistance increased only under CAF-P CM. OSCC cells specifically expressed insulin-like growth factor binding protein 3 (*IGFBP3*) after co-culture with CAF-D. Meanwhile, *IGFBP3*-knockdown OSCC cells acquired cisplatin resistance in CAF-D CM. *IGFBP3* expression was promoted by GATA-binding protein 1 (*GATA1*), a transcription factor targeted by miR-876-3p, which was enriched only in CAF-P-derived EV. Treatment with CAF-P EV carrying miR-876-3p antagomir decreased cisplatin resistance compared to control miRNA-carrying CAF-P EV. On comparing the staining intensity between cisplatin-sensitive and -insensitive tissues from OSCC patients, there was a positive correlation between *IGFBP3* and *GATA1* expression and cisplatin sensitivity in OSCC tissues from patients.

**Conclusion:** These results provide insights for overcoming cisplatin resistance, especially concerning EVs within the tumor microenvironment. Furthermore, it is anticipated that the expression levels of *GATA1* and miR-876-3p, along with *IGFBP3*, could aid in the prediction of cisplatin resistance.

## Introduction

More than 50% of patients with oral squamous cell carcinoma (OSCC), the most common type of head and neck squamous cell carcinoma (HNSCC), die within five years 1, 2. Chemotherapy is the most commonly used treatment for advanced or recurrent OSCC 3. Platinum-based chemotherapy is used to treat a wide of solid tumors 4. Cisplatin, one of the most common platinum compounds, is utilized alone or in combination, being part of the first-line treatment of approximately 50% of all cancer patients 5-7. However, 70-80% of patients with recurrent OSCC are resistant to cisplatin, which leads to poor clinical outcomes 8.

During the past decade, the role of the tumor microenvironment (TME) in tumor progression and chemoresistance has received considerable attention 9-14. As major cellular components of the tumor stroma, cancer-associated fibroblasts (CAFs) react to various cues and stimuli within their surrounding environment, thus significantly influencing chemotherapy 15, 16. A growing body of research suggests that CAFs promote OSCC progression by secreting growth factors and remodeling the extracellular matrix 17, 18. Notably, extracellular vehicle (EV) released from CAFs increase the chemoresistance of various cancer types 19, 20. EV participates in intercellular communication through the transfer of intracellular cargos such as proteins and miRNAs 21-23. In addition to tumor cell EV-derived miRNAs 24, miRNAs from CAF EV promotes drug resistance 16, 25, 26. However, the specific molecular mechanism through which CAFs modulate anticancer drug resistance, particularly in OSCC, is not well understood.

We previously reported that the primary CAFs from patients with OSCC could be functionally divided into two distinct groups 18. Those of the CAF-P (cancer-associated fibroblasts-Promoting properties) group promote OSCC progression and invasion, whereas CAF-D (Cancer-Associated Fibroblasts-Delaying properties) cells from other patients exhibited no significant effect or delayed cancer progression 18. In the present study, CAF-P and CAF-D showed differential regulation of chemosensitivity in OSCC. IGFBP3 (insulin-like growth factor binding protein 3), which increases cisplatin sensitivity, was significantly downregulated only under CAF-P co-culture. IGFBP3 is a secretory glycoprotein that modulates the mitogenic activity of insulin-like growth factor 1 receptor (IGF1R) 27, and inhibits angiogenesis *via* suppression of VEGF 28. Several studies have reported that lower IGFBP3 levels were associated with a greater risk of various cancers 29-31.

IGFBP3-overexpressing lung cancer cells showed higher sensitivity to cisplatin, resulting in attenuated cancer progression 32-34. With IGFBP3 downregulation in cisplatin-resistant cells, IGF-1 signaling through IGF-1R is maximized, stimulating metabolism, proliferation, and survival through the Ras-Raf-mitogen activated protein kinase (MAPK) and PI3K-PDK/AKT-TOR-S6K pathways 32, 33. Ectopic *IGFBP3* expression in breast cancer cells enhanced radiosensitivity *via* increasing the ratio of pro-apoptotic to anti-apoptotic members of the Bcl-2 family 35. Furthermore, in esophageal cancer cells, IGFBP3 promoted radiosensitivity by suppressing transition from G0/G1 to S phase, perhaps though influencing Smad3 and retinoblastoma protein (Rb) phosphorylation 36. Meanwhile, the upregulation of IGFBP3 in OSCC cells reduced radiosensitivity by promoting DNA repair 37. On the contrary, IGFBP3 was reported to promote radiosensitivity and chemosensitivity in OSCC cells *via* the positive regulation of ROS production 38. To date, few studies on the association of IGFBP3 with cisplatin resistance in OSCC have considered the TME context, a crucial aspect. Therefore, we sought to compare the capacity of the aforementioned CAF types for promoting OSCC progression through cisplatin resistance, with a particular focus on the role that the CAF-derived EV plays in the regulation of *IGFBP3* expression.

## Results

### CAF-P or CAF-D differentially affect cisplatin sensitivity in OSCC cells

**Figure 1:**
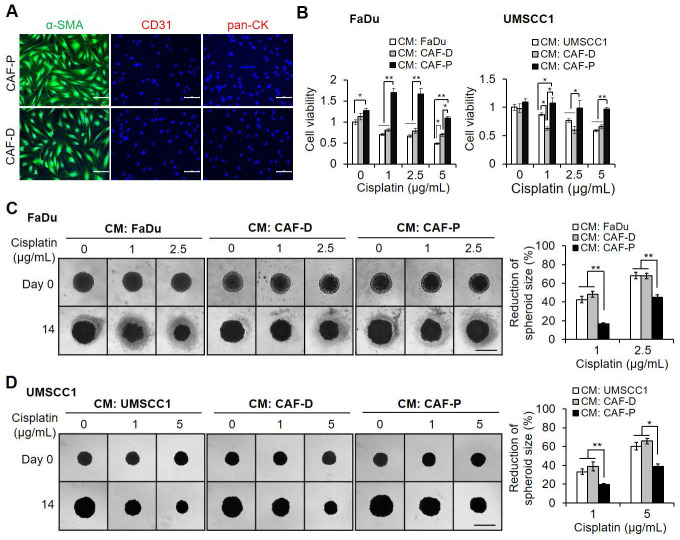
** Effect of CAF-P or CAF-D CM on cisplatin efficacy in OSCC cells. (A)** Primary fibroblasts were seeded in 6well plates containing cover slides and were immunostained with antibodies specific for fibroblasts (α-SMA), endothelial (CD31), and epithelial (pan-CK) cells. **(B)** After culturing OSCC cells in 96 well plates, the medium was changed with CM from CAF-D or CAF-P cells. After 16 h, cisplatin was treated for another 24 h, followed by MTT analysis. CMs from FaDu or UMSCC1 was used as controls. The spheroid formation with FaDu **(C)** and UMSCC1 **(D)** was observed in 96 well U-bottom ultra-low attachment plate for two days, followed by medium change with each CM. After cisplatin treatment for 14 days, spheroids were imaged using phase-contrast microscopy, and the size (surface area) was measured *via* Cell^3^iMager. Each experimental group consisted of eight spheroids, and a representative image is shown. The reduction of spheroid size is indicated by the relative percentage of size with cisplatin treatment compared with the size of vehicle treatment for 14 days. Results were presented as the mean ± standard deviation of three experiments. *p < 0.05; **p < 0.01. Scale bars: **A** 100 μm; **C-D** 500 μm.

Primary cultured CAF-P and CAF-D cells were stained with specific markers for CAFs, endothelial, and epithelial cells. Only CAF marker staining was positive (Figure 1A). In 2D cell culture, CAF-P CM significantly increased cisplatin resistance in both FaDu and UMSCC1 cells compared with CM from the control cell lines or CAF-D (Figure 1B). When spheroid size was monitored up to 14 days, the results were similar to those observed in 2D culture. CM from CAF-D CM showed a similar effect on cisplatin efficacy relative to FaDu or UMSCC1 CM as controls. However, CAF-P CM significantly increased cisplatin resistance in spheroids (Figure 1C-D). Collectively, the reduction in spheroid size at each cisplatin concentration relative to vehicle control was significantly decreased in CAF-P CM compared with that in CM from control OSCC cells or CAF-D.

### IGFBP3 was significantly upregulated in FaDu spheroids co-cultured with CAF-D

**Figure 2:**
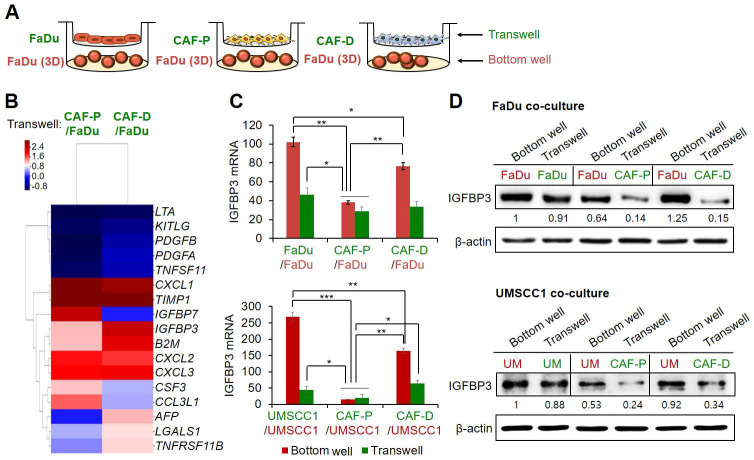
** Secretome antibody array in FaDu-CAF-P CM vs. FaDu-CAF-D CM. (A)** Antibody microarray with each CM was performed using a Cytokine profiling antibody array with six replicates per antibody. **(B)** Heatmap of differentially abundant factors in the secretome among the matched groups (fold change of > 2 and *p*-value of < 0.05). Array results of each CM derived from FaDu-CAF-P or FaDu-CAF-D were normalized to the FaDu-FaDu CM. **(C-D)** After co-culture of FaDu and UMSCC1 with each CAF-P and CAF-D cell, at the same condition presented in **A**, OSCC spheroids and CAF cells were collected from bottom well and Transwell, respectively. mRNA and protein expression of *IGFBP3* were compared using qPCR and western blot analysis. Results represent the mean ± standard deviation of three experiments. *p < 0.05; **p < 0.01; ***p < 0.005.

To further assess the differential effects of the two CAF types on cisplatin efficacy, we co-cultured FaDu spheroids (bottom well) with FaDu, CAF-P, or CAF-D cells for 48 h in a Transwell, respectively (Figure 2A). Each CM was subjected to antibody array analysis. As shown in Figure 2B, several secretory factors were differentially abundant between these CMs. Secreted proteins whose levels increased or decreased by more than 1.7-fold under FaDu-CAF-D CM relative to FaDu-CAF-P CM are summarized in Table S3. IGFBP3 levels were significantly increased under co-culture with CAF-D compared with those under CAF-P co-culture. To further confirm mRNA and protein expression of* IGFBP3,* the two OSCC cell lines were co-cultured with each CAF type under identical conditions, as shown in Figure 2A. At both levels, *IGFBP3* expression was higher in OSCC cells co-cultured with CAF-D cells (Figure 2C-D). FaDu-FaDu and UMSCC1-UMSCC1 co-culture were used as controls. As opposed to mRNA expression, the protein levels of *IGFBP3* showed no remarkable difference between the bottom wells or Transwells in the FaDu and UMSCC1 control conditions. Therefore, we investigated whether *IGFBP3* influences the cisplatin resistance associated with different CAF groups.

### Positive correlation between* IGFBP3* expression and cisplatin sensitivity in OSCC cells

**Figure 3:**
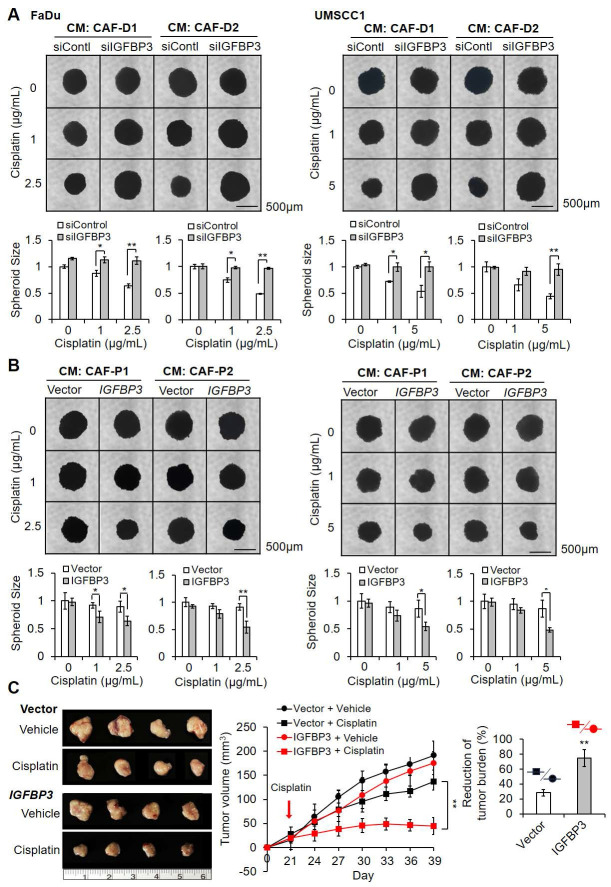
** Effect of *IGFBP3* on the cisplatin sensitivity in CMs from CAF-D or CAF-P. (A)** FaDu and UMSCC1 spheroids were formed for two days. After transfection with *siIGFBP3* for 24 h, the medium was replaced with CAF-D CM. After 16 h, cisplatin was added for another 14 days. **(B)** After transfection with a pCMV3-ORF-*IGFBP3* vector in spheroids for 24 h, the medium was replaced with each CM. After 16 h, cisplatin was added for another 14 days. The pCMV3 vector was used as a control. Spheroids were imaged *via* phase-contrast microscopy, and the size (surface area) was measured with Cell^3^iMager. Each experimental group comprises eight spheroids, and a representative image is given. Results were presented as the mean ± standard deviation of three experiments. **(C)** FaDu spheroids (< 400 μm in diameter) were prepared in 96 well plates. Overall, 50 FaDu spheroids (approximately 5 × 10^5^ cells) transfected with the overexpression vector were co-injected with the same number of CAF-P cells into the right and left backs of mice. After 20 days, cisplatin (2.5 mg/kg) or DMSO (0.1% v/v in PBS) vehicle control was intraperitoneally injected 2 times a week and sacrificed on the 19^th^ day after cisplatin administration. Tumor volume was measured using a caliper till sacrifice. The reduction of tumor burden represents the percentage of xenograft size reduction under cisplatin treatment compared with that under the vehicle-treated control vector or *IGFBP3*-overexpressed group. *p < 0.05; **p < 0.01. Scale bars: **A-B** 500 μm.

To determine the effect of *IGFBP3* on cisplatin sensitivity in OSCC cells cultured in CAF-D CM, we compared cisplatin efficacy after transfection with *siIGFBP3*. First, the knockdown efficiency of *siIGFBP3* in 2D culture with CM from control OSCC cells was maintained for two days, based on mRNA and protein expression (Figure S1A). *IGFBP3* knockdown decreased the cisplatin sensitivity of both cells (Figure S1B). The knockdown efficiency of *siIGFBP3* in OSCC cells under two different CAF-D CM was also maintained for two days, based on mRNA expression (Figure S1C). *siIGFBP3* pretreatment with CAF-D CMs induced a significant increase in chemoresistance in both cells (Figure S1D). Additionally, the cisplatin sensitivity of spheroids derived from the two cell lines was significantly decreased for 14 days following *siIGFBP3* pretreatment under CAF-D CM conditions (Figure 3A). FaDu and UMSCC1 spheroids exhibited reduced *IGFBP3* expression at the mRNA and protein level for 14 days after *siIGFBP3* transfection (Figure S1E).

We then investigated cisplatin efficacy in *IGFBP3*-overexpressing OSCC cells in CAF-P CM. *IGFBP3* mRNA and protein expression was significantly increased for two days of culture with control OSCC cell CM (Figure S2A). *IGFBP3* overexpression increased the cisplatin sensitivity of both cells under the same conditions (Figure S2B). In OSCC cells under two different CAF-P CM, overexpression was maintained for two days, based on mRNA expression (Figure S2C). When *IGFBP3* was overexpressed in FaDu and UMSCC1 and cells were then treated with cisplatin while under culture with CAF-P CM, cisplatin sensitivity was significantly enhanced compared with that in the control vector group (Figure S2D). Spheroids derived from the two cell lines exhibited a significant increase in cisplatin sensitivity for 14 days under the same experimental conditions (Figure 3B). In 3D spheroids, the overexpression vector was effective under CAF-P CM for 14 days, at both the mRNA and protein level (Figure S2E).

To further evaluate the effect of *IGFBP3* overexpression on cisplatin sensitivity, we subcutaneously transplanted *IGFBP3*-overexpressing spheroids with primary CAF-P cells into mice for 20 days and then induced xenograft formation. Upon cisplatin administration for another 19 days, tumor size in the *IGFBP3* overexpression group was significantly reduced compared with that in the control vector group, suggesting enhanced cisplatin sensitivity (Figure 3C). No significant difference in tumor size was observed between the *IGFBP3*-overexpression vector and control vector groups. The xenograft tissues were counterstained with H&E. A strong signal was shown for Ku80, a human cell-specific marker, indicating Ku80-positive fibroblast cells (Figure S3A). Immunostaining with the anti-KRT13 antibody revealed squamous epithelial xenografts in both groups (Figure S3B). The mRNA and protein expression of *IGFBP3* in the overexpression group was remarkably higher than that in the control vector group (Figure S3C).

### CAF-P-derived EV increased cisplatin resistance

**Figure 4:**
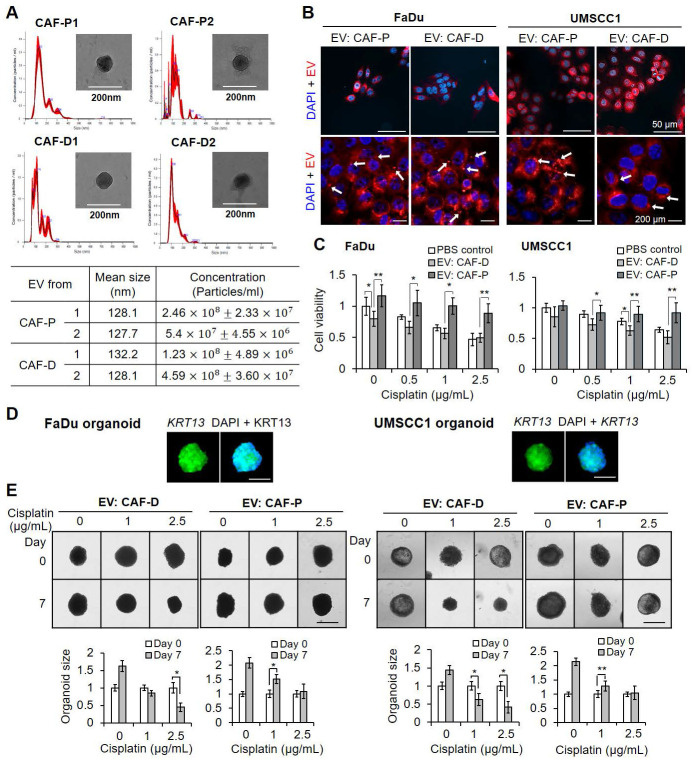
** Effect of CAF-derived EV on cisplatin resistance. (A)** Characterization of EV derived from CAF-P and CAF-D cells was performed *via* NTA. TEM images of EV revealed round-shaped vesicles. The mean size of EV and concentrations were presented. **(B)** EVs were pre-stained with lipid tracer dye DiD (red), followed by incubation with cells for 24 h at 37 °C. Confocal microscopy was used to detect EV internalization into cells. The white arrows indicate EV internalized into cells. **(C)** EVs (1 ͯ 10^7^ particles/mL, MOI = 100) were incubated with cells for 24 h, followed by cisplatin treatment for another 24 h. MTT assay was performed to compare cell viability. **(D)** OSCC organoids derived from mouse xenografts formed with FaDu or UMSCC1 cells were stained with an antibody against KRT13, a representative squamous epithelial cell marker. **(E)** EVs (MOI = 100) were incubated with organoids in 24 well plate for 16 h, followed by cisplatin treatment for another seven days. The organoid size was monitored using a Nikon ECLIPSE Ti microscope. Each experimental group consisted of approximately five organoids per 24 well plate, and a representative image is presented. Results represent the mean ± standard deviation of 2-3 experiments. *p < 0.05; **p < 0.01. Scale bars: **D-E** 100 μm.

We then evaluated whether EV from CAF-P and CAF-D had differential effects on cisplatin sensitivity. No substantial difference in size or concentration was observed between the two groups (Figure 4A). After staining of EV and treatment of OSCC cells, we employed confocal microscopy to determine whether EVs were taken up by the cells. As shown in Figure 4B, EVs were distributed inside both cells. Overall, there was no significant difference in cisplatin sensitivity between negative PBS control and CAF-D-derived EV. However, CAF-P-derived EV enhanced cisplatin resistance in both cells (Figure 4C). To confirm that cisplatin resistance caused by CAF-P CM was due to EV, we used CM pretreated with *GW4869,* a potent *inhibitor* of *EV* production. GW4869-pretreated CAF-P CM relieved cisplatin resistance compared with DMSO-pretreated CAF-P CM (Figure S4A). To determine whether *IGFBP3* protein was expressed in CAFs, and loaded onto CAF-derived EV, western blot analysis was performed on protein samples extracted from isolated EVs. As shown in Figure S4B, *CD63*, a representative EV marker, showed a strong signal, but no discernible signal for *IGFBP3* was noted in EVs derived from the two CAF types. This observation supports the results in Figure 2D. Organoids derived from FaDu or UMSCC1 xenografts were characterized by immunostaining for KRT13, a representative squamous epithelial marker (Figure 4D). The enhanced cisplatin resistance due to CAF-P-derived EV was also observed in spheroids (Figure S4C) and organoids (Figure 4E) formed by FaDu or UMSCC1 cells.

### CAF-P-derived EV enhanced cisplatin resistance in OSCC organoids through the downregulation of IGFBP3

**Figure 5:**
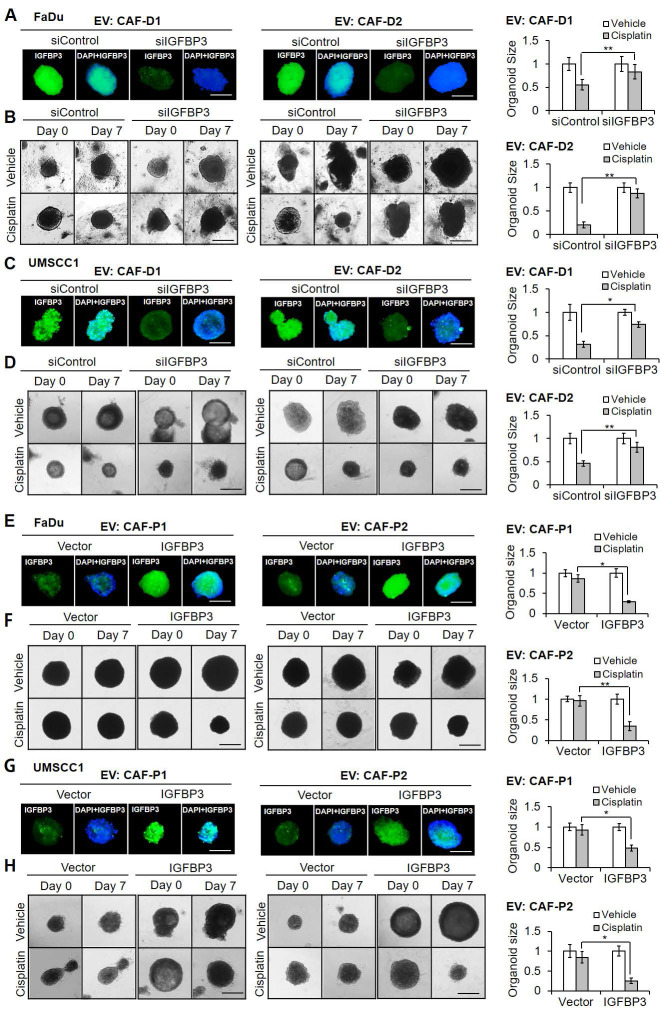
** Effect of CAF-derived EV on *IGFBP3*-dependent cisplatin sensitivity in OSCC organoids. (A, C)** Organoids derived from FaDu or UMSCC1 xenografts were transfected with *siIGFBP3* for 24 h, followed by CAF-D EV treatments (MOI = 100) for another 16 h. After seven days of cisplatin treatment, *IGFBP3* protein expression was compared *via* IF staining. **(B, D)** The organoid size was monitored at the same condition using a Nikon ECLIPSE Ti microscope. **(E, G)** Organoids were transfected with an *IGFBP3* overexpression vector for 24 h, followed by CAF-P EV treatments (MOI = 100) for another 16 h. After seven days of cisplatin treatment, *IGFBP3* protein expression was compared *via* IF staining. **(F, H)** The organoid size was monitored at the same condition. Results were presented as the mean ± standard deviation of three independent experiments. *p < 0.05; **p < 0.01. Scale bars: **A-H** 100 μm.

We sought to evaluate whether there is a positive correlation between* IGFBP3* expression level and cisplatin sensitivity in OSCC cells treated with EV derived from CAF subgroups. First, we compared the effect of *IGFBP3* gain- and loss-of-function on cisplatin sensitivity in FaDu and UMSCC1 organoids in fresh medium without any EV treatment as a control. As shown in Figure S5A, *siIGFBP3* pretreatment with cisplatin increased cisplatin resistance significantly in both organoids. However, there was no significant difference in cisplatin sensitivity with *IGFBP3* overexpression vector pretreatment (Figure S5B). To further evaluate whether EV derived from CAFs affect cisplatin sensitivity in organoids *via IGFBP3*, we transfected organoids with *siIGFBP3* or the overexpression vector, followed by EV pretreatment with cisplatin. *siIGFBP3* transfection in organoids cultured with CAF-D-derived EV caused a remarkable decrease in *IGFBP3* protein levels (Figure 5A, C) and mRNA expression (Figure S5C) after seven days. Furthermore, cisplatin sensitivity decreased significantly in both organoids cultured with CAF-D EV under siIGFBP3 effect (Figure 5B, D). Meanwhile, exogenous *IGFBP3* overexpression recovered cisplatin sensitivity with CAF-P EV treatment (Figure 5F, H). *IGFBP3* overexpression in organoids was effective for seven days, based on protein (Figure 5E, G) and mRNA levels (Figure S5D).

### CAF-P-derived EV downregulated *IGFBP3 via GATA1* transcriptional regulation

**Figure 6:**
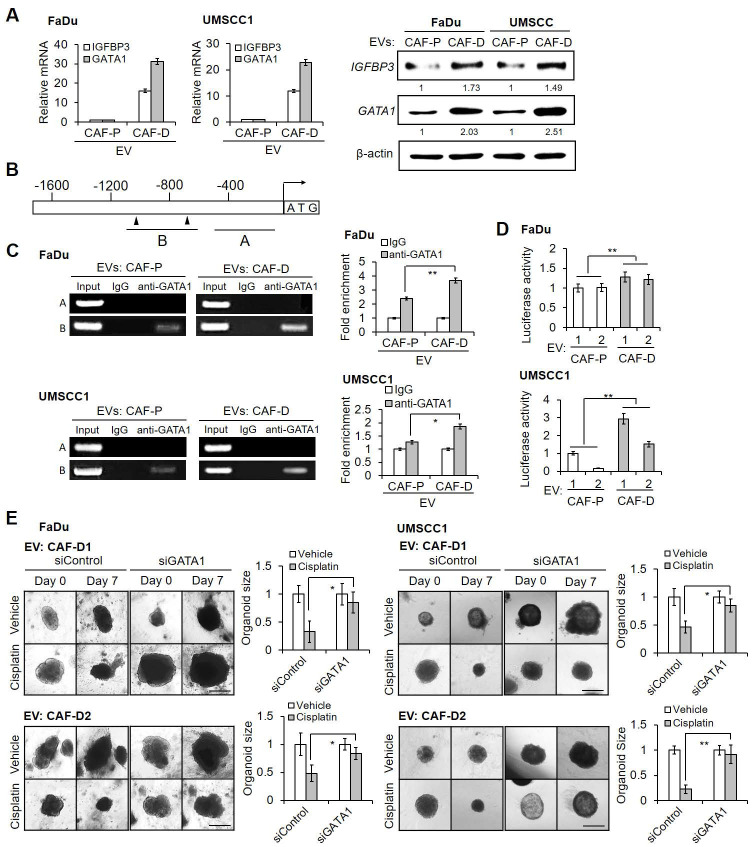
** CAF-P EV-dependent decrease of *IGFBP3 via GATA1* downregulation. (A)** mRNA and protein expression of *IGFBP3* and *GATA1* was evaluated following treatment with EV (MOI = 100) from CAF-P or CAF-D in OSCC cells for two days. **(B)** Schematic presentation of promoter regions for the *IGFBP3* gene showing the consensus *GATA1* binding sites, as determined *via* ChIP analysis (black arrowheads). **(C)** The chromatin of FaDu cells was immunoprecipitated using a *GATA1* antibody, and the resulting immunoprecipitants were analyzed using PCR to detect the consensus sequence of the *IGFBP3* promoter. PCR products were compared using gel electrophoresis, and a representative image is shown. Input and IgG were used as controls. **(D)** Luciferase activity was measured in OSCC cells pretreated with EV from two different CAF-Ps (1, 2) or CAF-Ds (1, 2) for 16 h, followed by transfection with each target plasmid. After 24 h, a dual luciferase assay was performed. **(E)** Organoids derived from OSCC xenografts were transfected with *siGATA*1 for 24 h, followed by two different CAF-D EV treatment (MOI = 100) for another 16 h. After seven days of cisplatin treatment, the organoid size was monitored using a Nikon ECLIPSE Ti microscope. Results were presented as the mean ± standard deviation of three experiments. *p < 0.05; **p < 0.01. Scale bars: **E** 100 μm.

In light of *IGFBP3* expression exhibiting substantial downregulation under CAF-P but not CAF-D CM, we explored the molecular mechanism underlying the observed differential *IGFBP3* expression. We identified *GATA1* as a highly probable transcription factor driving *IGFBP3* expression by using Harmonizome 3.0 website and searching for the GATA consensus binding sequence. OSCC cells pretreated with EV derived from CAF-D cells exhibited a parallel upregulation of the mRNA and protein expression levels of *IGFBP3* and *GATA1* as compared to cells pretreated with EV from CAF-P cells (Figure 6A). We performed a ChIP assay in OSCC cells treated with CAF-P- or CAF-D-derived EV, using an anti-GATA1 antibody. Figure 6B shows a schematic *IGFBP3* promoter region containing the consensus GATA1-binding sites (-1055 to -695). GATA1-bound promoter sequences increased significantly in CAF-D EV-treated cells compared with those in CAF-P EV-treated cells (Figure 6C). To further investigate possible transcriptional regulation by GATA1, luciferase reporter constructs were generated with the promoter region of *IGFBP3*. As shown in Figure 6D, *IGFBP3* promoter activity increased significantly following treatment with EV derived from CAF-D compared with that under treatment with CAF-P-derived EV in both cell lines.

We evaluated whether the expression of *GATA1*, as a transcription factor driving *IGFBP3*, would affect cisplatin resistance. First, OSCC cells were treated with *siGATA1*, and *GATA1* suppression was confirmed at the mRNA and protein levels (Figure S6A). In both cell lines, *siGATA1* pretreatment increased chemoresistance compared with the control siRNA pretreatment (Figure S6B). Furthermore, *siGATA1* significantly increased cisplatin resistance under treatment with CAF-D-derived EV (Figure S6C), showing the same effect as observed for CAF-P-derived EV treatment. In spheroids (Figure S6D) and organoids (Figure S7), the siRNA-mediated suppression of *GATA1* expression under CAF-D-derived EV treatment was confirmed *via* qPCR and IF staining. In both spheroids (Figure S6E) and organoids (Figure 6E), *siGATA1* pretreatment significantly enhanced the cisplatin resistance observed with CAF-D EV. These results revealed that GATA1 acts as a transcriptional regulator of *IGFBP3* expression, thus modulating cisplatin efficacy in an epistatic manner.

### hsa-miR-876-3p in CAF-P-derived EV induced cisplatin resistance *via* downregulation of GATA1

**Figure 7:**
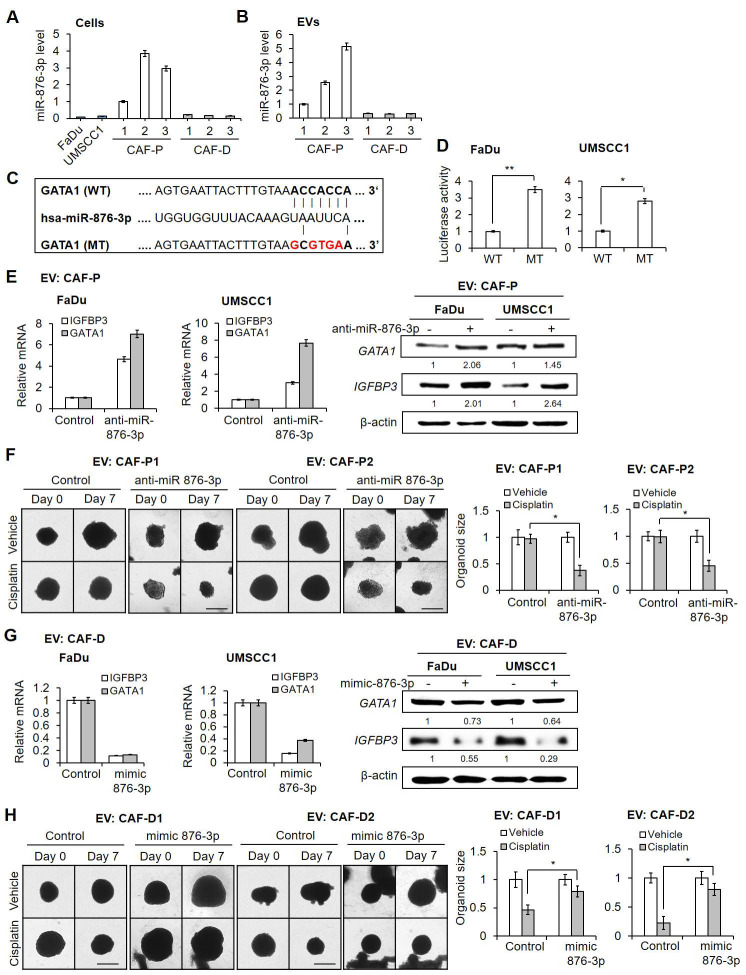
** Effect of hsa-miR-876-3p derived from CAF-P EV on cisplatin sensitivity. (A-B)** hsa-miR-876-3p mRNA level in OSCC cells, CAFs, and EVs, were compared. **(C)** Predicted miRNA binding site within the 3′-UTR of *GATA1* mRNA is shown. Mutations in the *GATA1* 3′-UTR are shown in red. Luciferase reporter constructs were generated with the wild-type (WT) and mutant (MT) 3′-UTRs of *GATA1*. **(D)** Dual luciferase reporter activity demonstrating the target relationship between miR-876-3p and *GATA1* mRNA. The activity was normalized to that of Renilla luciferase. The activity was measured in FaDu and UMSCC1 cells transfected with the WT and MT 3′-UTR *GATA1* luciferase constructs. **(E)** OSCC cells were transfected with anti-miR-876-3p and treated with CAF-P EV (1 ͯ 10^7^ particles/mL, MOI = 100) for 24 h. mRNA and protein expression of *IGFBP3* and *GATA1* in OSCC cells was analyzed *via* qPCR and western blot analysis. **(F)** FaDu organoids were transfected with anti-miR-876-3p and treated with CAF-P EV for 24 h, followed by cisplatin treatment for another seven days. **(G)** OSCC cells were transfected with mimic miR-876-3p and treated with CAF-D EV (1 ͯ 10^7^ particles/mL, MOI = 100) for 24 h. mRNA and protein expression of *IGFBP3* and *GATA1* in OSCC cells was analyzed *via* qPCR and western blot analysis. **(H)** FaDu organoids were transfected with mimic miR-876-3p and treated with CAF-D EV for 24 h, followed by cisplatin treatment for another seven days. The organoid size was monitored using a Nikon ECLIPSE Ti microscope. Results were presented as the mean ± standard deviation of three experiments. * p < 0.05; **p < 0.01. Scale bars: **F, H** 100 μm.

To further narrow down the molecular mechanism through which CAF-P-derived EV suppresses GATA1, we searched for miRNAs that regulate *GATA1* expression. Targetscan (http://www.targetscan.org/) yielded hsa-miR-876-3p as the candidate with the highest probability. miR-876-3p levels in OSCC, primary CAF-P, and CAF-D cells were then compared, as were those in EV from each cell type. As shown in Figure 7A, CAF-P cells exhibited significantly higher miR-876-3p levels than OSCC cells and CAF-D cells. Additionally, EV derived from CAF-P cells carried more miR-876-3p than CAF-D-derived EV (Figure 7B). The putative interaction between miR-876-3p and the 3*'-*UTR sequences of *GATA1* mRNA is shown in Figure 7C. Luciferase reporter constructs generated with the wild-type (WT) and mutant (MT) 3′*-*UTRs of *GATA1* were introduced, whereafter cells were treated with CAF-P-derived EV. As shown in Figure 7D, luciferase activity in OSCC cells transfected with wild type (WT) constructs was significantly lower than that in cells with mutant (MT) constructs. Furthermore, when OSCC cells were transfected with anti-miR-876-3p, the mRNA and protein expression of *GATA1* and *IGFBP3* increased significantly under CAF-P-derived EV treatment (Figure 7E). Anti-miR-876-3p enhanced cisplatin sensitivity in organoids derived from FaDu, even with CAF-P EV treatment (Figure 7F). In contrast, mimic miR-876-3p decreased the mRNA and protein expression of *GATA1* and *IGFBP3* following CAF-D-derived EV treatment (Figure 7G). Resistance to cisplatin was significantly enhanced under the same condition in FaDu organoids (Figure 7H). Collectively, CAF-P EV was enriched for miR-876-3p, which inhibited *GATA1* expression in OSCC cells, resulting in increased cisplatin resistance *via IGFBP3* downregulation.

### Xenografts formed by anti-miR-876-3p-transfected FaDu spheroids and CAF-P cells showed increased cisplatin sensitivity

**Figure 8:**
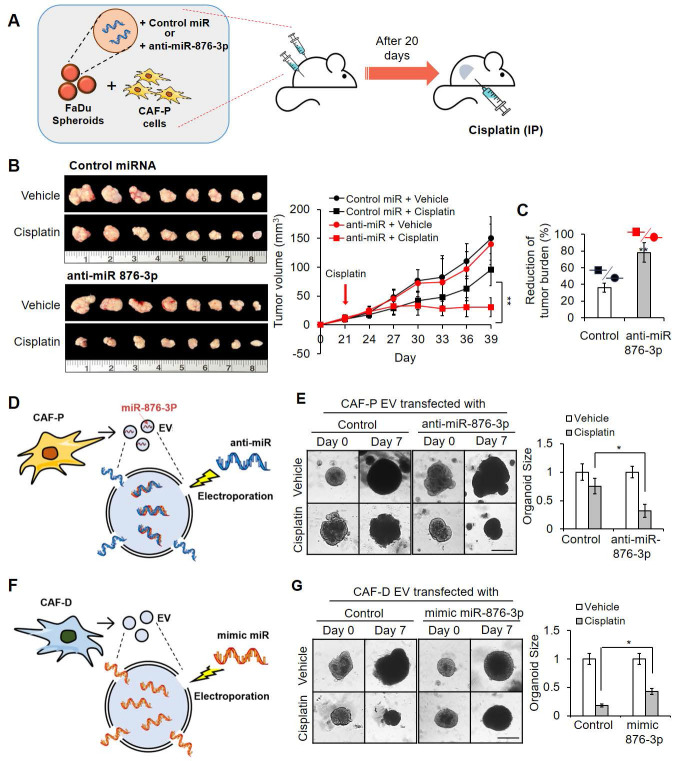
** Effect of antagomir or mimic miRNA for miR-876-3p on cisplatin sensitivity in OSCC. (A)** FaDu spheroids (< 300 μm in diameter) were prepared in 96 well plates, and transfected with anti-miR-876-3p for 16 h. Overall, 50 spheroids (approximately 5×10^5^ cells) were co-injected with the same number of CAF-P cells into the right and left backs of mice. After 20 days, cisplatin (2.5 mg/kg) or DMSO (0.1% v/v in PBS) vehicle control was intraperitoneally injected 2 times a week and sacrificed on the 19^th^ day after cisplatin administration. **(B)** Tumor volume was measured using a caliper till sacrifice. **(C)** The reduction of tumor burden represents the percentage of xenograft size reduction under cisplatin treatment compared with that under the control miRNA-transfected or anti-miR-876-3p-transfected group. **(D)** CAF-P-derived EVs were loaded with anti-miR-876-3p *via* electroporation, followed by RNase treatment and re-purification. **(E)** CAF-P EVs carrying anti-miR-876-3p were transfected in organoids (MOI = 100) for 16 h, followed by cisplatin treatment for seven days. **(F)** CAF-D-derived EVs were loaded with mimic miR-876-3p *via* electroporation, followed by RNase treatment and re-purification. **(G)** These EVs were treated in organoids (MOI = 100) for 16 h, followed by cisplatin treatment for seven days. The organoid size was monitored using a Nikon ECLIPSE Ti microscope. Results were presented as the mean ± standard deviation of three independent experiments. *p < 0.05; **p < 0.01. Scale bars: **E, G** 100 μm.

To confirm the effect of miR-876-3p on cisplatin resistance, we subcutaneously transplanted anti-miR-876-3p-carrying FaDu spheroids with primary CAF-P cells into mice for 20 days (Figure 8A). Upon cisplatin administration for another 20 days, tumor size in the anti-miR-876-3p-transfected group was significantly reduced compared with that in the control miRNA-carrying group, suggesting enhanced cisplatin sensitivity (Figure 8B-C). No significant difference in tumor size was observed between the anti-miR-876-3p and control miRNA groups. mRNA and protein expression of *IGFBP3* and* GATA1* in the anti-miR-876-3p group was remarkably higher than that in the control miRNA group (Figure S8A). We observed a strong signal for Ku80, a human cell-specific marker, in the xenografts (Figure S8B). Immunostaining with the anti-KRT13 antibody revealed squamous epithelial xenografts in both groups (Figure S8C).

### EVs carrying miR-876-3p antagomir or mimic alleviated cisplatin sensitivity changes in OSCC

To further evaluate whether miR-876-3p in CAF-P-derived EV affects cisplatin resistance, OSCC spheroids and organoids were pretreated with anti-miR-876-3p-transfected CAF-P-derived EV (Figure 8D) or mimic miR-876-3p-transfected CAF-D-derived EV (Figure 8F) prior to cisplatin. *GATA1* and *IGFBP3* protein levels were both upregulated and downregulated in organoids subjected to anti-miR-876-3p-transfected or mimic miR-876-3p-transfected EV treatment, respectively (Figure S9A). CAF-P-derived EV carrying anti-miR-876-3p decreased cisplatin resistance relative to those carrying control miRNA in both FaDu organoids (Figure 8E) and OSCC spheroids (Figure S9B). In contrast, CAF-D-derived EV carrying mimic miR-876-3p enhanced cisplatin resistance compared with control miRNA-transfected CAF-D EV (Figure 8G and Figure S9C). The mRNA expression of *GATA1* and *IGFBP3* was upregulated in spheroids treated with anti-miR-876-3p-transfected EV and downregulated in those treated with mimic miR-876-3p-transfected EV, for 14 days (Figure S9C, S9E). Collectively, miR-876-3p, specifically enriched in CAF-P-derived EV, inhibited GATA1-dependent *IGFBP3* expression, thus promoting cisplatin resistance.

### Correlation between *IGFBP3* and *GATA1* expression and cisplatin sensitivity in clinical OSCC tissues

**Figure 9:**
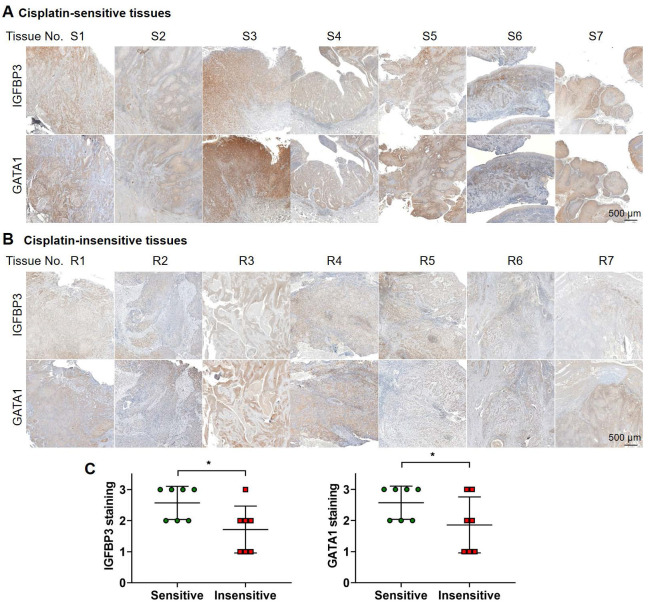
**IHC analysis of OSCC tissues form patients.** Tissues from patients with OSCC showing (**A**) cisplatin-sensitive and (**B**) cisplatin-insensitive tissues were stained with anti-IGFBP3 and anti-GATA1 antibodies. **(C)** The level of IGFBP3 on each specimen was scored as 0, 1, 2, and 3 (0 = negative, 1 = weak, 2 = intermediate, and 3 = strong) according to its staining intensity. *p < 0.05.

We investigated the relationship between *IGFBP3* and *GATA1* expression and cisplatin sensitivity in patients with OSCC. We categorized patients based on their response to cisplatin monotherapy, either sensitive or insensitive by their tumor size changes and survival. Figure 9 displays staining results from seven tissue samples in each group. On comparing the staining intensity between cisplatin-sensitive and -insensitive group, tissues from cisplatin-sensitive patients exhibited a robust signal to *IGFBP3* and *GATA1*, whereas tissues from cisplatin-resistant patients displayed a comparatively weaker response. These results affirm the positive correlation between *IGFBP3* and *GATA1* expression and cisplatin sensitivity in OSCC tissues, supporting our findings.

## Discussion

CAFs express various factors that shape the TME, including tumor-promoting as well as tumor-suppressive molecules 39. Giguelay *et al.* previously showed that CAF subpopulations exhibit functional heterogeneity 40. We previously identified two functional CAF types based on their differential effect on cancer progression in OSCC tissue samples from patients 18. In the present study, we determined whether and the mechanisms through which these two CAF types differentially affect cisplatin resistance, with a particular focus on CAF-derived EV. We observed CAFs that promote tumor progression more vigorously (CAF-P) induce cisplatin resistance, whereas CAFs with lesser cancer-promoting properties (CAF-D) do not. This differential effect was attributed to the EV secreted from respective CAF types. Ultimately, hsa-miR-876-3p, present at high levels only in the CAF-P-derived EV, downregulated *GATA1* in cancer cells. Consequently, *IGFBP3*, one of the target genes of GATA1, is also suppressed, resulting in cisplatin resistance.

*IGFBP3* is upregulated in certain chemo- or radiosensitive cervical, ovarian, and lung cancer cell lines compared with levels in matched resistant cells 33, 34, 41. Furthermore, treatment of lung cancer cells with recombinant human IGFBP3 confers sensitivity to cisplatin 32. In HNSCC, IGF modulation *via* IGFBP3 was proposed as an adjuvant therapy for overcoming drug resistance 42. However, data on the interplay between cancer cells and the TME with regard to *IGFBP3*-regulated cisplatin efficacy are currently lacking. Our present results revealed that CAFs strongly promote cancer progression and selectively suppress *IGFBP3* expression *via* GATA1 transcriptional downregulation, which results in cisplatin resistance in OSCC. *GATA1* was recently reported as involved in tumorigenesis, cancer progression 43, 44, and carboplatin resistance in ovarian cancer 45. To date, there has been no data on the effect of *GATA1* on cisplatin resistance.

Tumor cells can become resistant to drugs either by direct contact with cells in the TME or through communication mediated by proteins like cytokines and growth factors 46-48. Recently, it has been recognized that EVs play a vital role in intercellular communication in the TME by carrying proteins and nucleic acids. Specifically, EVs released in TME contain miRNAs that, upon uptake by other cells, can impact the expression of their genes, influencing chemosensitivity 49, 50. In our study, as two types of CAFs regulate *IGFBP3* expression differently in OSCC cells, we anticipated that EV-derived miRNAs could play a crucial role. Hence, we focused on EVs among the various communication molecules within the TME. The role of CAFs in tumor drug resistance has attracted increasing attention, and they play an important role in the regulation of tumor drug resistance mediated by EVs 26, 51-53. Notably, CAF EV-derived miR-21, miR-27a, miR-106, miR-146a, or miR‐522 promote chemoresistance 54-58. Qin *et al.* reported that CAF EV-derived miR-196a confers cisplatin resistance in HNSCC, and its high levels in plasma are clinically correlated with poor overall survival and chemoresistance 59. In this study, we identified CAF-P EV-derived miR-876-3p as the functional molecule that confers cisplatin resistance in OSCC. Meanwhile, the level of miR-876-3p in CAF-D-derived EV was rather low, enabling higher *IGFBP3* expression and greater cisplatin efficacy when compared with CAF-P-derived EV. A previous study suggested that miR-876-3p enhanced cisplatin sensitivity in gastric cancer 60, which contrasts our findings. Furthermore, miR-876-3p restricted the stem cell-like features of gastric cancer cells by targeting TMED3 61. In colon cancer, miR-876-3p exerted significant inhibitory effects on cell proliferation, migration, and invasion, thus exhibiting a tumor-suppressive role 62. Collectively, further evaluation is required to elucidate whether the differential effects of CAF-D and CAF-P on OSCC progression are also due to miR-876-3p.

Various efforts have recently been made to address tumor drug resistance using EVs 26, 63. While most studies focus on EVs as carriers for delivering drugs, there is also interest in exploring the potential of overcoming drug resistance by miRNAs within EVs. When miR-214 inhibitor was loaded onto EVs which were then incubated with gastric cancer cells, the inhibitor was successfully introduced into cancer cells and reversed drug resistance 64, 65. EV-mediated delivery of functionally active miRNA inhibitor by intravenous injection reduced tumorigenicity of breast cancer in mouse model, suggesting the concept of using EV as efficient nanovehicles for RNA-based therapeutics 66. Hu *et al.* suggested that exosomal miR-1229 activated VEGF pathway, and treatment with an antagomir against miR-1229 impaired tubulogenesis of HUVECs and inhibited tumor growth in xenograft model 67. This data suggest that extracellular antagomir can affect gene expression in tumor cells in the xenograft TME, same as our experimental method, which was obtained by directly injecting antagomir into mice. In our study, transplanting the miR-876-3p antagomir along with CAF-P cells significantly reduced cisplatin resistance of OSCC xenografts. Considering the well-established role of EVs by delivering their contents to surrounding target cells, it is anticipated that manipulation of EV miRNAs with specific antagomir or mimic miRNA can play a pivotal role in various physiological processes in TME.

In this study, we provide extensive evidence that CAFs with enhanced cancer-promoting properties (CAF-P) induce cisplatin resistance *via* exosomal miR-876-3p secretion. Nevertheless, the present study has some limitations. The effect of antagomir-fed EV on cisplatin efficacy should be investigated in a xenograft model. The association between CAF properties, *IGFBP3* expression, and cisplatin efficacy in primary organoids derived from the tissues of patients with OSCC is another aspect that remains to be addressed. In particular, we examined *IGFBP3* and *GATA1* expression in both cisplatin-sensitive and -insensitive OSCC tissues within the scope of this study. However, only the small number of patients receiving cisplatin monotherapy led to each group comprising only seven tissue samples. The current results indicate that the expression patterns of *IGFBP3* and *GATA1* in both cisplatin-sensitive and -insensitive tissues from OSCC patients strongly align with our findings. Moving forward, it is crucial to expand our research through retrospective studies, incorporating a larger number of patient tissues suitable for this study. Additionally, it is necessary to compare the levels of miR-876-3p in plasma EVs among patients with varying response to cisplatin.

In conclusion, our results suggest that CAFs, which markedly promote cancer progression, specifically induce cisplatin resistance in OSCC *via* EV. miR-876-3p was identified as the critical factor in CAF-P-derived EV, which downregulates *IGFBP3* by suppressing *GATA1* in OSCC cells. Thus, miR-876-3p, *IGFBP3*, and *GATA1* represent promising biomarkers for cisplatin resistance in OSCC chemotherapy. In addition, EV-based therapies targeting drug resistance genes can potentially overcome cisplatin resistance in cancer patients. The current study highlights the differential drug resistance-conferring capacity of CAFs against the same cancer cells, thus emphasizing the decisive role that TME plays in anticancer drug resistance. In addition, modulating EV with specific antagomir or mimic miRNA may hold promise as a viable treatment.

## Materials and Methods

### Reagents

The UMSCC1 (mouth floor tumor, RRID: CVCL_7707) cell line was obtained from Merck KGaA (Darmstadt, Germany), and the FaDu (hypopharyngeal tumor, RRID: CVCL_1218) cell line was obtained from the American Type Culture Collection (ATCC). Dulbecco's Modified Eagle's medium (DMEM), fetal bovine serum (FBS), and penicillin-streptomycin were acquired from Invitrogen (USA). 3-[4, 5-dimethyl-2-thiazolyl]-2, 5-diphenyl-2H-tetrazolium bromide (MTT), Trizol (15596-018), GW4869 (D-1692), and cisplatin (232120) were purchased from Sigma-Aldrich (Merck, Germany). TOPreal™ SYBR Green qPCR PreMIX was purchased from Enzynomics (RT500S, South Korea). Rabbit anti-IGFBP3 (10189-2-AP, RRID: AB_2123233), anti-GATA1 (10917-2-AP, RRID: AB_2108279), and anti-KRT13 (10164-2-AP, RRID: AB_2134679) were purchased from Proteintech (USA). Mouse anti-β-actin (sc-47778, RRID: AB_626632) was purchased from Santa Cruz Biotechnology (USA). Rabbit anti-α-SMA (ab5694, RRID: AB_2223021), anti-CD31 (ab225883, RRID: AB_570940), and mouse anti-pancytokeratin (pan-CK) antibodies (ab7753, RRID: AB_306047) were purchased from Abcam (USA). Anti-mouse (Alexa Fluor 488 conjugate, A-11001, RRID: AB_2534069) and anti-rabbit (Alexa Fluor 633 conjugate, A-21070, RRID: AB_2535731) secondary antibodies were obtained from Invitrogen (USA). Mimic (MC12886) and antagomir (AM12886) of miR-876-3p were purchased from Thermo Fisher Scientific (USA). IGFBP3 (sc-39587) and GATA1 (sc-35452) siRNA mixture containing 2-3 specific oligonucleotides were purchased from Santa Cruz Biotechnology (USA).

### Primary fibroblast culture from fresh OSCC tissues

Tumor tissues for fibroblast culture were obtained from four OSCC patients at Kyungpook National University Hospital. Patient information for primary CAFs is presented in supplemental Table S1. Stroma adjacent to the tumor was carefully isolated by the pathologist, cut into the smallest pieces possible in sterile DMEM, and cultured with 10% FBS.

### Two-dimensional (2D) and three-dimensional (3D) cell culture

The OSCC cells were cultured in DMEM containing 10% FBS and 1% penicillin-streptomycin solution, at 37 °C in a 5% CO_2_ humidified atmosphere. Cells were tested for contamination every two months using the BioMycoX^®^ Mycoplasma PCR detection kit (CellSafe, South Korea). For 3D spheroid formation, cells (4000 cells/well) were seeded into a 96-well U-bottom ultra-low attachment plate (7007, Corning Incorporated, USA) and cultured for 2-3 days to form spheroids with uniform sizes in each well (> 300 μm in diameter). The spheroid size was determined by measuring the surface area of each group containing 6-8 spheroids using a Cell^3^iMager scanner CC-5000 (SCREEN Holdings, Japan). Each spheroid's surface area was the same at the beginning of the experiments, within the 5% error range.

### Preparation of conditioned medium (CM), EV isolation, and staining

Primary CAFs were cultured in DMEM supplemented with 10% FBS and 1% penicillin/streptomycin for 24 h. The medium was replaced with serum-free DMEM, and cells were incubated for an additional 48 h. The medium was then filtered through a 0.45 μm filter, followed by storage at -20 °C until use. For EV isolation, CM was centrifuged at 2000 ×*g* for 30 min at 4 °C. The supernatant was then transferred to a new tube, combined with Total Exosome Isolation reagent (Thermo Fisher Scientific, USA). EVs were isolated *via* centrifugation at 10,000 × *g* and 4 °C for 1 h. The EV-containing pellets were resuspended in PBS. EV yield was determined using a nanoparticle tracking analysis system (Nanosight NS300, UK). To acquire transmission electron microscopy (TEM) images of EV, we prepared three grids (200 mesh, 01800-F, Ted Pella Inc., USA.) coated with formvar/carbon per sample. The EV samples were dried on a grid and observed under an electron microscope (HT7700, Hitachi, Japan). For staining, EVs in 100 μL of PBS were incubated with 10 μM red fluorescent Lipophilic Tracer 1,1′-dioctadecyl-3,3,3′,3′-tetramethylindodicarbocyanine, 4-chlorobenzenesulfonate salt (DiD; Thermo Fisher Scientific, D7757, USA) for 1 h at 37 °C. To ensure EV incorporation into cells, cells plated on coverslips were incubated with stained EV (1 × 10^7^ particles/24 well/mL, MOI = 100) for 12 h. Cells were fixed with 4% PFA and washed with PBS. Nuclei were counterstained with DAPI (Vector Laboratories, USA) at a ratio of 1:1000 for 10 min, and the coverslip was mounted on a slide. After drying the slides, cells were visualized using a laser-scanning confocal microscope (LSM Zeiss 700; Carl Zeiss, Germany).

### Cell viability under cisplatin after pretreatment with CAF-derived CM or EV

Cells were seeded in 96-well plates at a density of 10,000 cells/well. On the next day, the medium was changed with CMs from CAF-P or CAF-D and incubated for 16 h. CMs from OSCC cells were used as controls. After incubation with cisplatin for another 24 h, cell viability was confirmed using the MTT assay. In 3D models, the effect of cisplatin was quantified based on the size difference of 3D spheroids (surface area) and organoids (average area). After spheroid formation in 96-well U-bottom ultra-low attachment plates, the medium was changed to CM or fresh medium containing EV. After 16 h, cisplatin was added to each well. DMSO (0.1% v/v in PBS) was used as the vehicle control. The experiment was conducted with eight spheroids per group, and the spheroids were monitored for 14 days using a phase-contrast microscope. In a 24-well plate, the experiment was conducted with five organoids per well as a group, and the size of organoids was determined after seven days using a Nikon ECLIPSE Ti microscope (5 × magnification). EVs were treated at multiplicity of infection (MOI) of 100, under all experimental conditions.

### Antibody microarray

To confirm the effect of CAF-P and CAF-D on cisplatin sensitivity, secretome analysis was performed using CM after co-culture with 3D-spheroids of FaDu cells for 48 h. An antibody array featuring 310 antibodies for profiling human cytokines, chemokines, and related biomarkers was used, with six replicates per antibody (Full Moon Biosystems, USA). For secretome screening, two types of biotin-label-based antibody arrays were combined to detect over 500 serum proteins. After dialysis of each sample, the primary amines were biotinylated using a biotin-labeling step (Pierce, USA). Biotin-labeled proteins were incubated with ASB600 (Full Moon Biosystems, USA) and AAH-BLG 507 (RayBiotech, USA) at 26 °C for 2 h. After incubation with Cy3-conjugated streptavidin, the signal was visualized using a slide scanner (Axon Instruments, GenePix 4000 B, USA). The fluorescence signal of each antibody spot was obtained from the fluorescence intensity of the antibody-stained area, and data from the arrays were normalized to positive markers. *p*-values were calculated using a two-sample Student's t-test. Candidate biomarkers were identified based on a *p*-value < 0.05 and fold-change > 2 adjusted by multivariate statistics. Data mining and graphic visualization were performed using the ExDEGA software (Ebiogen Inc., Korea).

### qPCR and protein expression analysis

Quantitative PCR was performed for mRNA expression analysis. RNA extraction, cDNA synthesis, and gene expression normalization were performed according to standard protocols. The primers used in qPCR are shown in supplementary Table S2. qPCR was performed in triplicate using an ABI 7500 real-time PCR system (Applied Biosystems, USA). Gene expression levels were normalized to *GAPDH*. The fold-change in gene expression level was calculated based on Δ cycle threshold (ΔCt) values.

The concentrations of total protein samples were determined. Equal amounts of total protein (20-40 µg) were separated *via* 8-10% sodium dodecyl sulfate-polyacrylamide gel electrophoresis (SDS-PAGE) and transferred to a nitrocellulose membrane. After blocking with 5% skim milk for 1 h, the membrane was incubated overnight at 4 °C with primary antibodies. β-actin served as the loading control. HRP-conjugated secondary antibodies were applied for 1 h at room temperature, and the blot was washed thrice in Tris-buffered saline containing 0.1% Tween 20. Protein bands were detected using enhanced chemiluminescence, and their relative intensities were analyzed using ImageJ software (National Institutes of Health, USA).

### Transfection of siRNA or overexpression vector

OSCC cells, spheroids, and organoids were transfected with siRNA mixture containing 2-3 specific oligonucleotides. More detailed information is presented in the Supplementary methods. The pCMV3-ORF-vector (Sino Biological Co., China) was used for exogenous overexpression at a final concentration of 100 ng/96 well or 500 ng/24 well. The empty pCMV3-ORF vector was used as a control.

### Organoid culture

Mouse xenografts derived from OSCC cells were used for organoid culture. Tissue processing and organoid culture were performed as described by Driehuis *et al.* 68, 69. Primary tissue pieces were washed with 45 mL of ice-cold complete medium (adDMEM/F12+++: advanced DMEM/F12 medium (Thermo Fisher Scientific, 12634-010, USA) supplemented with 1× GlutaMAX (Thermo Fisher Scientific, 35050-061), penicillin-streptomycin (Thermo Fisher Scientific, 15140-122, USA), 10 mM HEPES (Thermo Fisher Scientific, 15630-056, USA), and 100 μg/mL Primocin (Invivogen, ant-pm-1, USA). Tissue pieces were crushed into small fragments of 1-3 mm^3^ in a 10 cm cell culture dish using surgical scissors or scalpels. Minced tissue samples were digested for < 1 h incubations in TrypLE (Gibco, 12605-028, USA), and the contents were mixed every 10-15 min by vigorous shaking. When the mixture became cloudy, the remaining tissue fragments became disrupted. We pipetted up and down 20 times using a P1000 pipette. After centrifugation at 200 × g for 5 min at 4 °C, the pellets were resuspended in 10 mL of adDMEM/F12+++ medium and filtered using a 100 μm cell strainer. The samples were centrifuged one more time at 200 × *g* for 5 min at 4 °C, the supernatant was aspirated, and pellets were resuspended in cold BME (R&D Systems, 3533-005-02, USA). Approximately 50 μL droplets were plated on the bottom of preheated suspension culture plates (Greiner Bio-One, M9312, SG). After seeding, the plates were incubated at 37 °C for 30 min for BME to solidify. Prewarmed organoid medium (adDMEM/F12+++ containing 1× B27 supplement (Thermo Fisher Scientific17504-044, USA), 1.25 mM N-acetyl-L-cysteine (Sigma-Aldrich, A9165, USA), 10 mM nicotinamide (Sigma-Aldrich, N0636, USA), 50 ng/mL human EGF (PeproTech, AF-100-15, USA), 0.5 μM A83-01, 10 ng/mL human FGF10 (PeproTech, 100-26, USA), 5 ng/mL human FGF2 (PeproTech, 100-18B, USA), 1 μM prostaglandin E2 (Tocris Bioscience, 2296, UK), 0.3 μM CHIR 99021 (Sigma-Aldrich, SML1046, USA), 1 μM forskolin (R&D Systems, 1099, USA), 4% R-spondin, and 4% Noggin (both produced *via* the r-PEX protein expression platform at U-Protein Express BV) was subsequently added to the plate. The medium was changed every 2-3 days, and organoids were split once every 1-2 weeks. Cisplatin efficacy was monitored for seven days using a Nikon ECLIPSE Ti microscope (Nikon Imaging Japan Inc., Japan).

### Immunofluorescence (IF) staining of spheroids, organoids, and xenografts

IF staining was performed on cultured spheroids, organoids, and xenografts. The experiment was performed as described by Sarvestani *et al.* 70. Spheroids or organoids in Matrigel were released in PBS. Spheroids, organoids, and xenografts were fixed with 4% paraformaldehyde for 2 h at room temperature. After frozen sectioning, these were treated with blocking serum (5% serum in 1 × PBS plus 0.5% Triton-X100) for 1 h prior to overnight incubation with the primary antibodies. The next day, organoids were washed in PBS-0.5% Triton-X100 and incubated with secondary antibodies for 2 h. To counterstain nuclei, the organoids were suspended in a drop of VECTASHIELD mounting medium containing DAPI and loaded onto a cover-well imaging chamber for fluorescence microscopy. Organoids underwent all staining procedures in a slide chamber. Images were captured with a ZEISS Axio microscope (ZEISS Microscopy, Germany).

### Mouse xenograft model

To evaluate the effect of IGFBP3 on cisplatin efficacy in mouse xenografts, FaDu spheroids transfected with pCMV3-ORF-IGFBP3 or the control vector (50 spheroids per injection, approximately 5 × 10^5^ cells) were transplanted with the same number of CAF-P cells into the right and left dorsal sides of mice using a 22-gauge needle. To evaluate the effect of miR-876-3p derived from CAF-P EV on cisplatin efficacy in mouse xenografts, FaDu spheroids transfected with antagomir targeting miR-876-3p (50 spheroids per injection, approximately 5 × 10^5^ cells) were transplanted with the same number of CAF-P cells into the right and left dorsal sides of mice. After tumor formation, each group was randomly divided into two (n = 4 or n = 8 tumors per subgroup): cisplatin and vehicle injection control. Cisplatin (2.5 mg/kg) or vehicle control was intraperitoneally injected two times a week, and the mice were sacrificed on the 20^th^ day after cisplatin administration. Tumor volume was measured using calipers. We used cell spheroids in this *in vivo* experiment because the persistence of exogenous gene expression efficiency in 3D spheroids was far superior to that in 2D cells. Concerning tumor size, we followed the guidelines for sacrifice when the mouse weight was reduced by 20% or the tumor volume was ≥ 10 cm^3^.

### Immunohistochemical (IHC) analysis

Tissue sections obtained by paraffin-embedded blocks were used. Human tissue blocks were obtained from patients with OSCC who underwent biopsy or tumor resection for oral cancer treatment from 2016 to 2022 at the Kyungpook National University Dental Hospital. Patient information is presented in supplementary Table S4. After dewaxing, sections were blocked for 5 min, followed by incubation for 2 h at room temperature with specific primary antibodies (1:500-1:100). IHC staining was performed using the UltraTek Horseradish Peroxidase (HRP) Anti-olyvalent Kit (ScyTek Laboratories, USA); the chromogen used was 3,3-diaminobenzidine (Dako, USA). The image was acquired under light microscope at 40 × magnification. The level of IGFBP3 on each specimen was scored as 0, 1, 2, and 3 (0 = negative, 1 = weak, 2 = intermediate, and 3 = strong) according to its staining intensity.

### Chromatin immunoprecipitation (ChIP) and luciferase reporter assay

A chromatin Immunoprecipitation Kit from EpiGentek (P-2002-1, USA) and a Dual-Luciferase Reporter Assay Kit (Promega, E1910, USA) were used. All experiments were conducted according to manufacturer instructions. PCR primers employed for these experiments were listed in supplementary Table S2. FaDu and UMSCC cells were cultured and treated with CAF-P- or CAF-D-derived EV for 12 h. After crosslinking with paraformaldehyde for 20 min, the cell pellets were lysed, followed by sonication to shear DNA to an average fragment size of 200-1000 bp. The DNA fragments were mixed with GATA1 or IgG antibody and immunoprecipitated overnight at 4 °C. The precipitated chromatin fragments were purified and analyzed *via* RT-qPCR and PCR.

For the *IGFBP3* promoter luciferase assay, OSCC cells were cultured and treated with CAF-P- or CAF-D-derived EV for 12 h. Subsequently, the cells were transfected in triplicate with 800 ng of various artificial target plasmids using Lipofectamine 3000 (Thermo Fisher Scientific, USA). After 24 h, a luciferase assay using the Dual-Luciferase Reporter Assay Kit (Promega, USA) was performed according to the manufacturer's protocol. For the miR-876-3p-dependent luciferase assay, OSCC cells were co-transfected with the pGL3 plasmid containing wild-type or mutant *GATA1* 3′-UTR and mimic miR-876-3p using Lipofectamine 3000 (Invitrogen, L3000001, USA). After 48 h, luciferase activity was measured using the same methods.

### Electroporation of the mimic miRNA or antagomir into CAF-derived EV

Anti-miR or mimic miRNA (Life Technologies, USA) was added to the EV derived from CAF-P of CAF-D cells, respectively, to a final concentration of 300 pM. An equal amount of unrelated scrambled miRNA was used as the negative control. The mixture was transferred to a cooled 0.2-cm electroporation cuvette and electroporated at 150 V to 100 μF using a Gene Pulser II system (Bio-Rad Laboratories, USA). Electroporated EVs were treated with RNase A and purified using an ExoQuick-TC kit (System Biosciences, USA).

### Statistical analysis

All *in vitro* experiments were performed two or three times. Statistical parameters, including analysis of the* in vivo* results, are presented in the figure legends. All statistical analyses were conducted using the Origin v.8.0 (OriginLab, USA) and R software. One-way ANOVA and an unpaired t-test were used for the statistical testing of comparisons between three or more and two groups, respectively. The levels of secreted factors were compared between CAF-P and CAF-D co-culture conditions using the Mann-Whitney U test. A *p*-value < 0.05 was considered as statistically significant. Significant *p*-values are shown in each figure.

### Ethics approval and consent to participate

Human tissue specimens were used after receiving written informed consent from the patients, with approval from the Institutional Research Ethics Committee of Kyungpook National University Hospital (KNUH201704011) and adherence to the principles of the Declaration of Helsinki. All experimental protocols with mice followed the ARRIVE guidelines (Animal Research: Reporting of *In Vivo* Experiments) and were approved by the Animal Ethics Committee of Kyungpook National University (2017-94-2).

### Data Availability

The secretome antibody array data generated in this study are publicly available in Gene Expression Omnibus (GEO) at GSE229247.

## Supplementary Material

Supplementary figures and tables.

## Abbreviations

CAF-D: cancer-associated fibroblast-defense
CAF-P: cancer-associated fibroblast-promote
CM: conditioned medium
EV: extracellular vesicle
HNSCC: head and neck squamous cell carcinoma
IF: immunofluorescence
IHC: immunohistochemistry
3D: three-dimension
2D: two-dimension
TME: tumor microenvironment
