# Supplementary material for: Differential effect of cancer-associated fibroblast-derived extracellular vesicles on cisplatin resistance in oral squamous cell carcinoma via miR-876-3p
Source: Theranostics. 2024 Jan 1;14(2):460–79. doi: 10.7150/thno.87329 (PMC10758057; doi:10.7150/thno.87329)
Supplement: Supplementary file 1 — Supplementary figures and tables. [file thnov14p0460s1.pdf]

Supplementary Information

Supplementary Tables

Table S1. Clinicopathological parameters of OSCC patients whose cells were obtained for primary CAF culture

CAF	Age	Sex	Primary site	Differentiation	Lymph node Meta	TMN	Stage	Smoking	Drinking
P	48	F	Buccal mucosa	Moderate	Y	T1N1M0	III	Y	Y
	76	M	gingiva	Moderate	N	T4N0M0	IV	N	N
D	64	M	Mouth floor	Moderate	N	T2N0M0	II	N	N
	47	M	Ventral surface of tongue	Well	N	T1N0M0	I	Y	N

Table S2. PCR primers

Experiments		F/R	sequence (5'→3')
qPCR for mRNA expression	*IGFBP3*	Forward	TGCTAGTGAGTCGGAGGAAG
		Reverse	GGGTGGAACTTGGGATCAGA
	GATA1	Forward	CTACTACAGGGACGCTGAGG
		Reverse	ACAGTTGAGGCAGGGTAGA
	GAPDH	Forward	AGATCATCAGCAATGCCTCCTG
		Reverse	ATGGCATGGACTGTGGTCATG
miRNA binding assay	WT	Forward	TCGAGTGAATTACTTTGTAAACCACCA GAATTC GC
		Reverse	CACTTAACGAAACATTTGGTGGTCTTAAG CGCCGG
	MT	Forward	TCGAGTGAATTACTTTGTAAGCGtGAAGAATTC GC
		Reverse	CACTTAACGAAACATTCGCACTTCTTAAG CGCCGG
ChIP	A	Forward	CGCAGTGCCTTGGCTCCCTGA
		Reverse	GCAACCGGGGCACGCTGCTTG
	B	Forward	ACAGGAGTTACAGCGACCTCA
		Reverse	CTGACTTACATTTGGGTCTCAGG
Promoter Luciferase activity	1	Forward	AATTTGGTACCGGAGGTCTCACCG
		Reverse	AATTAGATCTGACGCCTGCAACCG
	2	Forward	AATTGGTACCCTTGTAGACGACAAGGTGA
		Reverse	AATTAGATCTCTGCTTGGCAGGCTCC

Table S3. Differentially abundant proteins determined *via* secretome antibody array

Gene Symbol	Antibody name	keyword	Fold change			p-value
			FaDu + CAP-P	FaDu + CAP-D	FaDu+ CAP-D /FaDu+ CAP-P	FaDu+ CAP-D /FaDu+ CAP-P
IGFBP7	IGF-BP7	Alternative splicing; Cell adhesion; Complete proteome; Direct protein sequencing; Disulfide bond; Glycoprotein; Growth factor binding; Immunoglobulin domain; Polymorphism; RNA editing; Reference proteome; Secreted; Signal	5.518	0.977	0.177	0.0000
CCL3L1	LD78beta	Chemotaxis; Complete proteome; Cytokine; Direct protein sequencing; Disulfide bond; Reference proteome; Secreted; Signal	2.963	1.453	0.491	0.0000
CXCL10	IP-10	3D-structure; Chemotaxis; Citrullination; Complete proteome; Cytokine; Direct protein sequencing; Disulfide bond; Inflammatory response; Reference proteome; Secreted; Signal	1.697	0.924	0.544	0.0368
TGFB2	TGF beta2	3D-structure; Alternative splicing; Aortic aneurysm; Chromosomal rearrangement; Cleavage on pair of basic residues; Complete proteome; Direct protein sequencing; Disease mutation; Disulfide bond; Glycoprotein; Growth factor; Mitogen; Polymorphism; Reference proteome; Secreted; Signal	0.982	0.535	0.545	0.0000
CCL2	MCP-1/ MCAF	3D-structure; Chemotaxis; Complete proteome; Cytokine; Direct protein sequencing; Disulfide bond; Glycoprotein; Inflammatory response; Pyrrolidone carboxylic acid; Reference proteome; Secreted; Signal	1.419	0.790	0.557	0.0000
SPP1	Osteopon-tin	3D-structure; Alternative splicing; Biomineralization; Cell adhesion; Complete proteome; Cytokine; Direct protein sequencing; Glycoprotein; Phosphoprotein; Polymorphism; Reference proteome; Secreted; Sialic acid; Signal	1.003	0.573	0.571	0.0000
B2M	Beta-2-Microglo-bulin	3D-structure; Amyloid; Amyloidosis; Complete proteome; Direct protein sequencing; Disease mutation; Disulfide bond; Glycation; Glycoprotein; Immunity; Immunoglobulin domain; MHC I; Pyrrolidone carboxylic acid; Reference proteome; Secreted; Signal	2.249	4.479	1.992	0.0000
IGFBP3	IGF-BP3	Apoptosis; Complete proteome; Direct protein sequencing; Disulfide bond; Glycoprotein; Growth factor binding; Phosphoprotein; Polymorphism; Reference proteome; Secreted; Signal	2.268	5.280	2.328	0.0004
AFP	AFP	3D-structure; Complete proteome; Copper; Direct protein sequencing; Disulfide bond; Glycoprotein; Metal-binding; Nickel; Polymorphism; Reference proteome; Repeat; Secreted; Signal; Sulfation	0.968	2.299	2.374	0.0037

Table S4. Clinicopathological parameters of OSCC tissues from patients used for IGFBP3 and GATA1 immunostaining

Response to cisplatin	Age Sex	Primary site	Differentiation	Lymph node Meta	TMN stage	Stage
S1	48 F	Buccal mucosa	Moderate	Y	pT1N1M0	III
S2	65 M	Buccal mucosa	Moderate	Y	pT3N2bM0	III
S3	56 M	Rt. Mn. post. area	Moderate	Y	pT4aN2bM1	IVa
S4	74 F	Lt. Mn. post. area	Moderate	Y	pT3N2bM0	III
S5	63 M	Lt. Mx. post. area	Moderate	N	cT3N0M0	III
S6	82 F	Palatal	Poor	N	cT2N0M0	II
S7	73 M	Buccal mucosa	Moderate	Y	cT4aN2bM1	IVa
R1	59 F	Lt. Mn. post. area	Moderate	N	pT4aN0M0	IVa
R2	59 F	Lt. Mn. post. area	Moderate	N	pT4aN0M0	IVa
R3	60 M	Mn. ant. area	Well	Y	pT4aN2bM0	IVa
R4	78 M	Lower lip	Moderate	Y	cT4N1M0	IVa
R5	53 M	Mouth floor	Well	Y	cT4aN2aM0	IVa
R6	76 M	Buccal mucosa	Moderate	Y	pT4aN2bM0	IVa
R7	64 M	Tongue	Well	Y	pT2N1M0	III

(c: clinical stage, p : pathologic stage)

Supplementary Figures

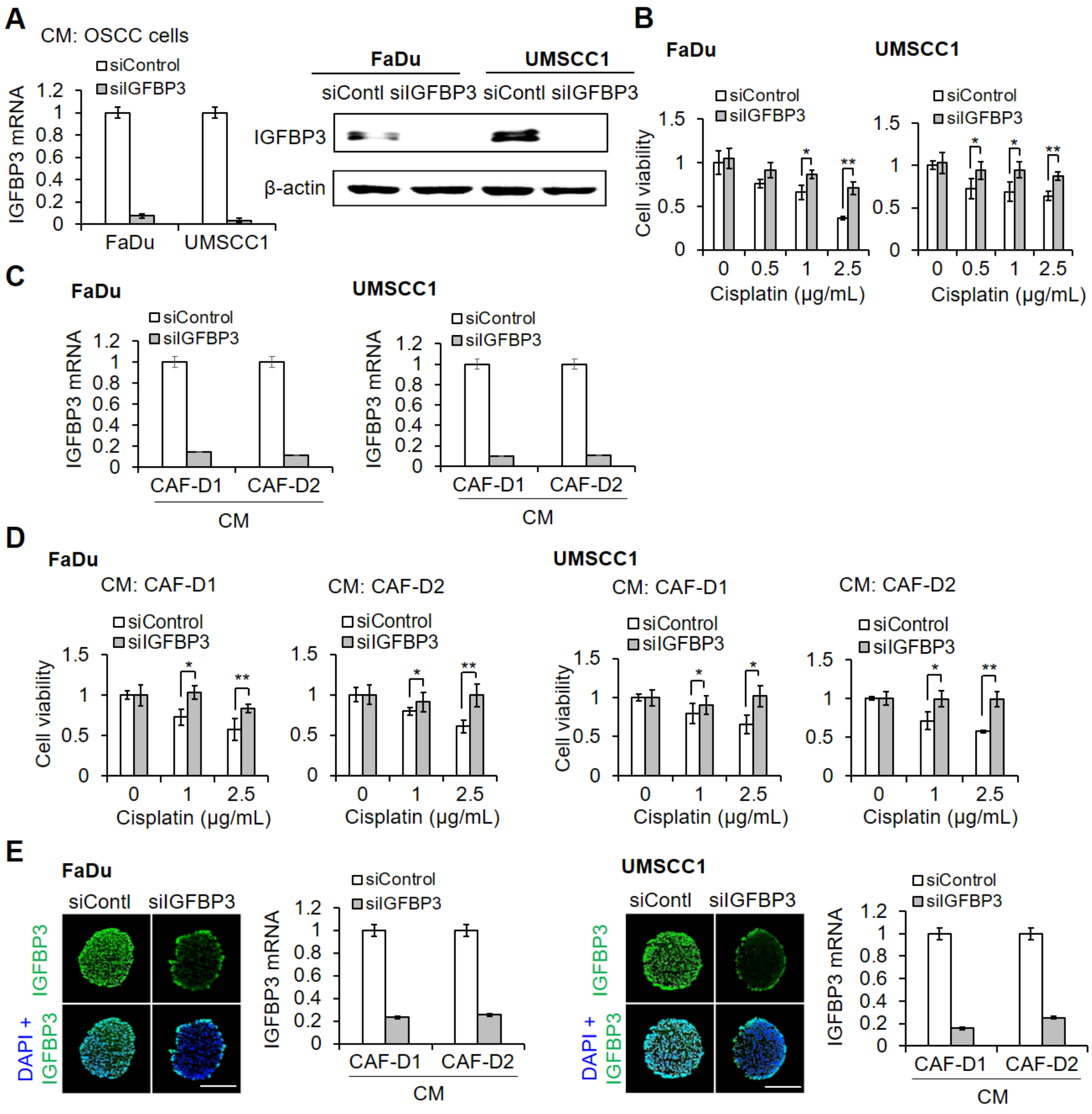

Figure. S1. Effect of *IGFBP3* knockdown on the cisplatin sensitivity of OSCC cells in CAF-D CM. (A) In control CM from FaDu or UMSCC1, OSCC cells were transfected with *siIGFBP3* for 24 h and treated with cisplatin for another 24 h. mRNA and protein expression of *IGFBP3* was analyzed. **(B)** Cell viability was evaluated under the same condition using MTT assay. **(C)** FaDu and UMSCC1 cells were transfected with *siIGFBP3* for 24 h, followed by medium change with each CAF-D CM. After 16 h, cisplatin was added for another 24 h, and *IGFBP3* mRNA expression was analyzed. **(D)** Cell viability was evaluated under the same condition using MTT assays. **(E)** FaDu and UMSCC1 spheroids were formed in 96-well U-

bottom ultra-low attachment plates for two days. After transfection with *siIGFBP3* for 24 h, the medium was replaced with CAF-D CM. After 16 h, cisplatin was added for another 14 days, and *IGFBP3* expression was analyzed at the mRNA and protein level *via* qPCR and IF staining, respectively. *$p < 0.05$; **$p < 0.01$. Scale bars: **E** 500 μm.

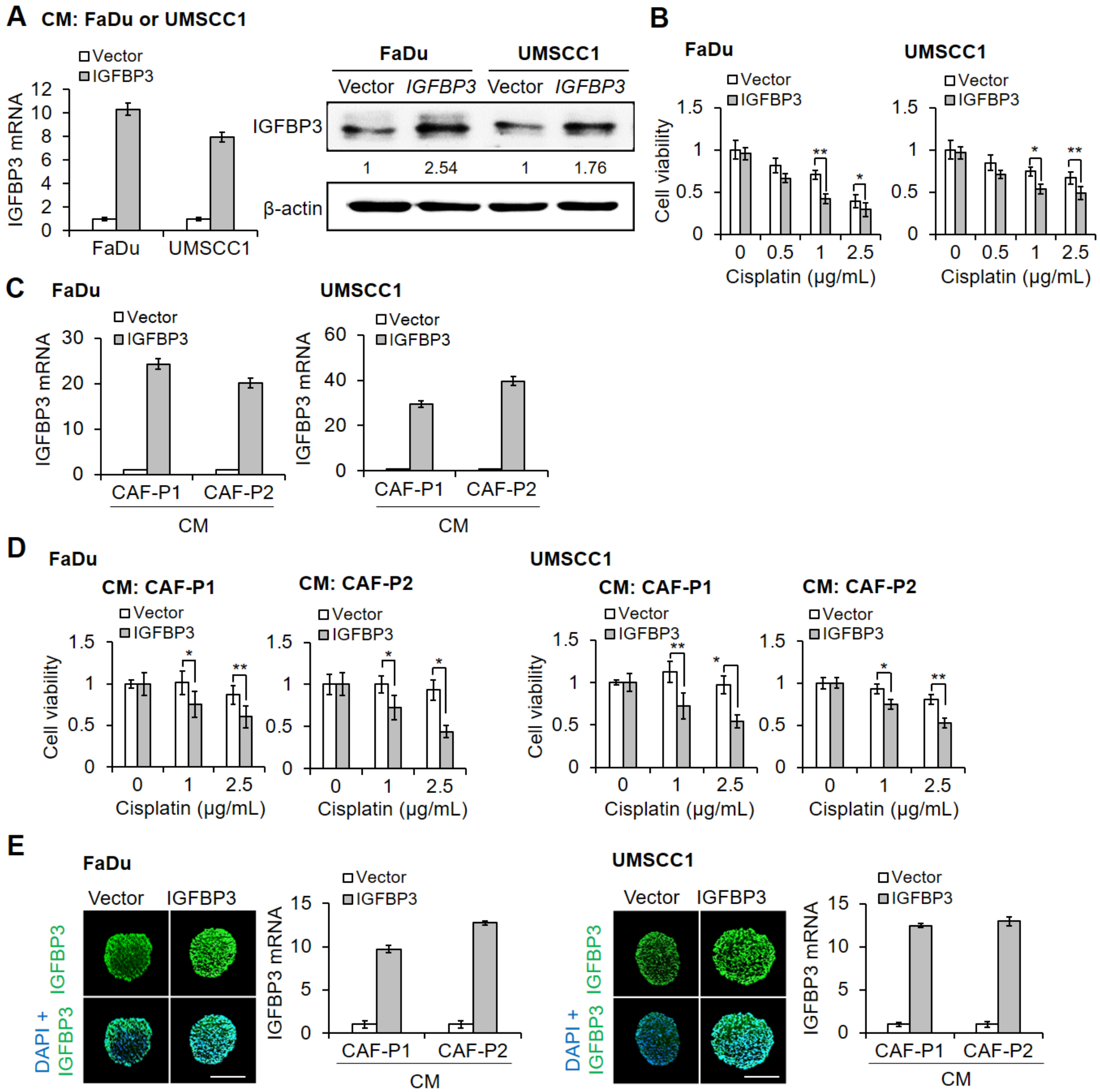

Figure S2. Effect of *IGFBP3* overexpression on cisplatin sensitivity in CAF-P CM. (A) In control CM from FaDu or UMSCC1, OSCC cells were transfected with an *IGFBP3* overexpression vector for 24 h and treated with cisplatin for another 24 h. The pCMV3 vector was used as a control vector. mRNA and protein expression of *IGFBP3* was analyzed. **(B)** Cell viability was evaluated using MTT assay under the same condition. **(C)** FaDu and UMSCC1 cells were transfected with an *IGFBP3* overexpression vector for 24 h, followed by medium change with each CAF-P CM. After 16 h, cisplatin was added for another 24 h, and *IGFBP3* mRNA expression was analyzed. **(D)** Cell viability was evaluated under the same condition using an MTT assay. **(E)** FaDu and UMSCC1 spheroids were formed in 96-well U-bottom ultra-low attachment plates for two days. After transfection with *IGFBP3* overexpression vector for 24 h, the medium was replaced with CAF-P CM. After 16 h, cisplatin was added for another 14 days, and *IGFBP3* expression was analyzed on the mRNA and

protein level via qPCR and IF staining, respectively. *p < 0.05; **p < 0.01. Scale bars: **E** 500 μm.

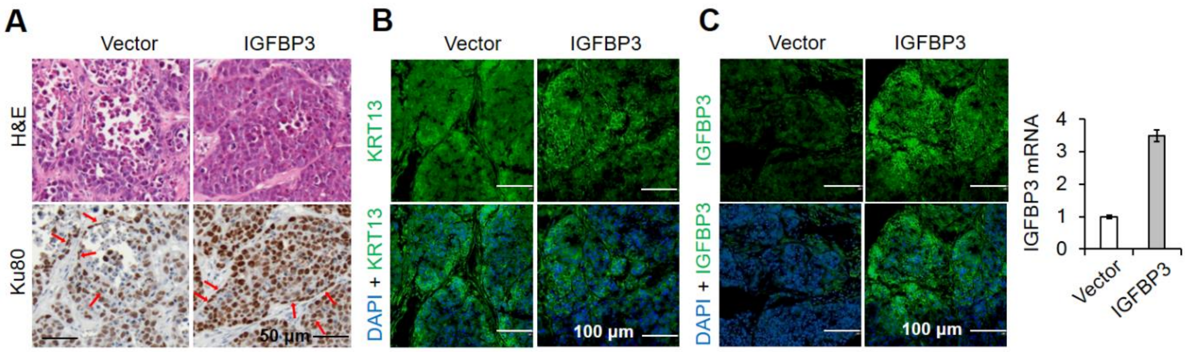

Figure S3. IHC and IF analysis of mouse tumor tissues. (A) Tissue sections were stained with anti-Ku80 antibody to determine whether these are derived from human cells. Representative Ku80-positive fibroblast cells were indicated with red arrows. Tissues were counterstained with H&E. **(B)** IF staining of mouse tumor tissues was performed with the KRT13 antibody to confirm squamous epithelial cell origin. **(C)** The mRNA and protein expression of *IGFBP3* in the xenografts were evaluated via qPCR analysis and IF staining, respectively. Results were presented as the mean ± standard deviation of three experiments. *p < 0.05; **p < 0.01.

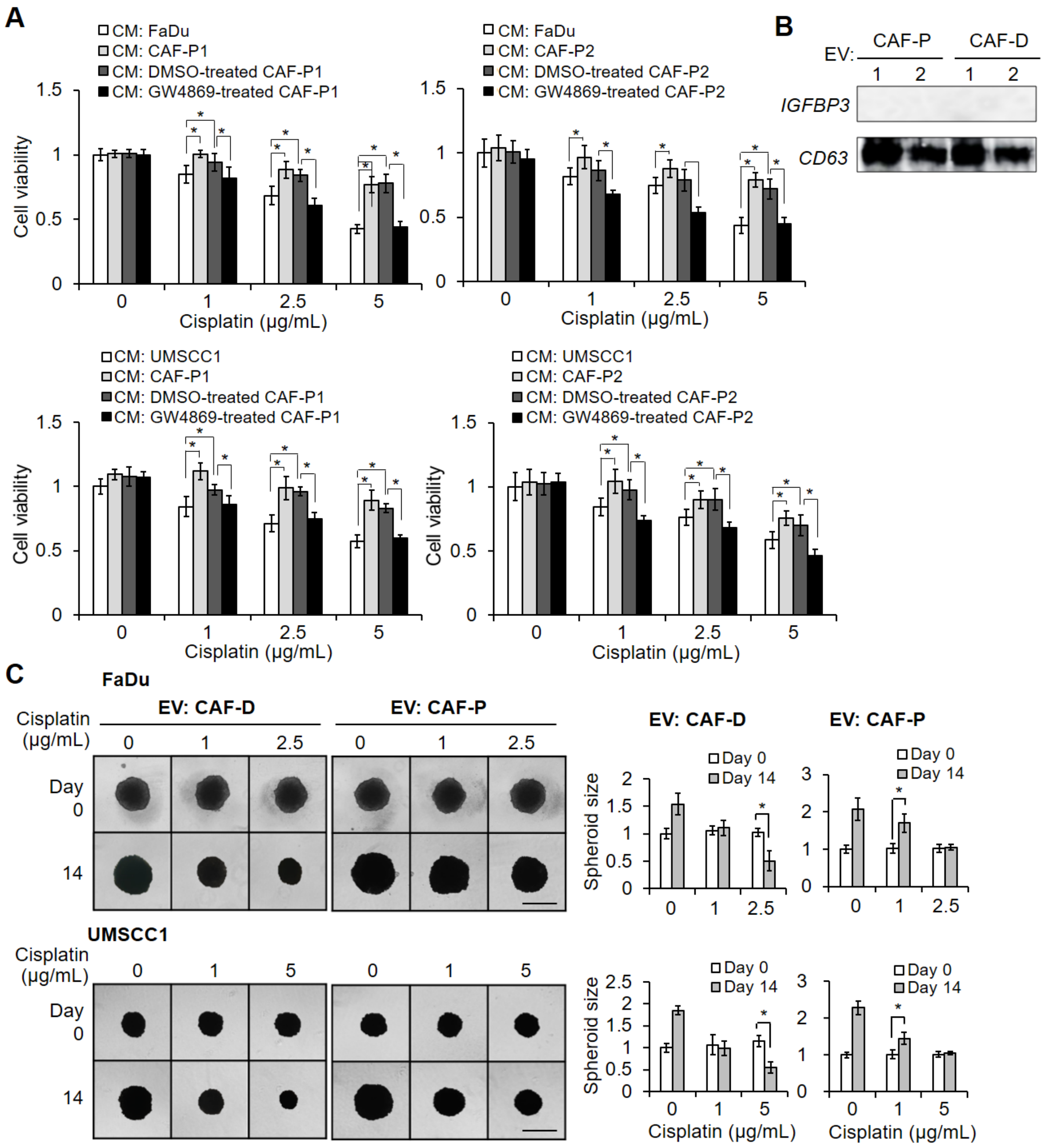

Figure S4. Effect of CAF-derived EV on cisplatin resistance in OSCC cells. (A) Two different CAF-P cell lines were cultured with GW4869, an inhibitor of EV production, for 2 days, followed by CM collection. Cell viability was compared between DMSO-treated CM and GW4869-treated CM after cisplatin treatment for 24 h. CM from OSCC cells were compared as controls. **(B)** EV-loaded IGFBP3 protein level was analyzed via western blot analysis. The level of CD63, a representative EV marker, was analyzed under the same condition. **(C)** Spheroids were formed in 96-well U-bottom ultra-low attachment plates for two days, followed by EV treatment for 16 h (1×10^7 particles/mL, MOI = 100). Cisplatin was added for another 14 days. Spheroids were imaged using phase-contrast microscopy, and their size (surface

area) was measured using Cell[3]iMager. Each experimental group consists of eight spheroids, and a representative image is shown. Results are presented as the mean ± standard deviation of three experiments. *p < 0.05. Scale bars: **C** 500 μm.

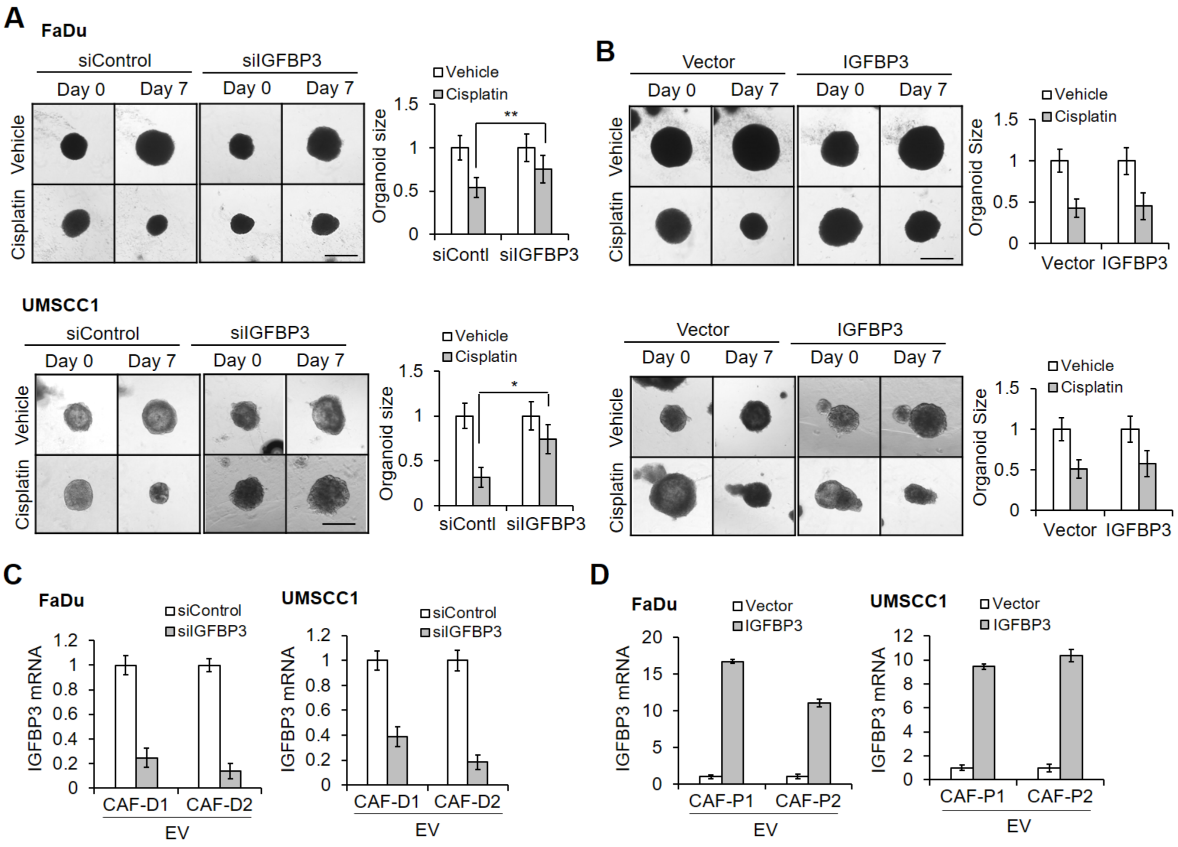

Figure S5. IGFBP3 siRNA- or overexpression vector-dependent cisplatin sensitivity and mRNA expression in organoids. (A) The effect of IGFBP3 knockdown on cisplatin sensitivity in FaDu and UMSCC1 organoids without any CM treatment was compared as a control. **(B)** The effect of IGFBP3 overexpression on cisplatin sensitivity in FaDu and UMSCC1 organoids without any CM treatment was compared as a control. **(C)** Organoids derived from FaDu or UMSCC1 xenografts were transfected with *siIGF*BP3 for 24 h, followed by CAF-D EV treatment (MOI = 100) for another 16 h. After seven days of cisplatin treatment, *IGFBP3* mRNA expression was compared via qPCR. **(D)** *IGFBP3* overexpression vector-pretreated organoids treated with cisplatin for seven days were analyzed for *IGFBP3* mRNA expression via qPCR. Results are presented as the mean ± standard deviation of three experiments. *p < 0.05; **p < 0.01. Scale bars: **A-B** 100 μm.

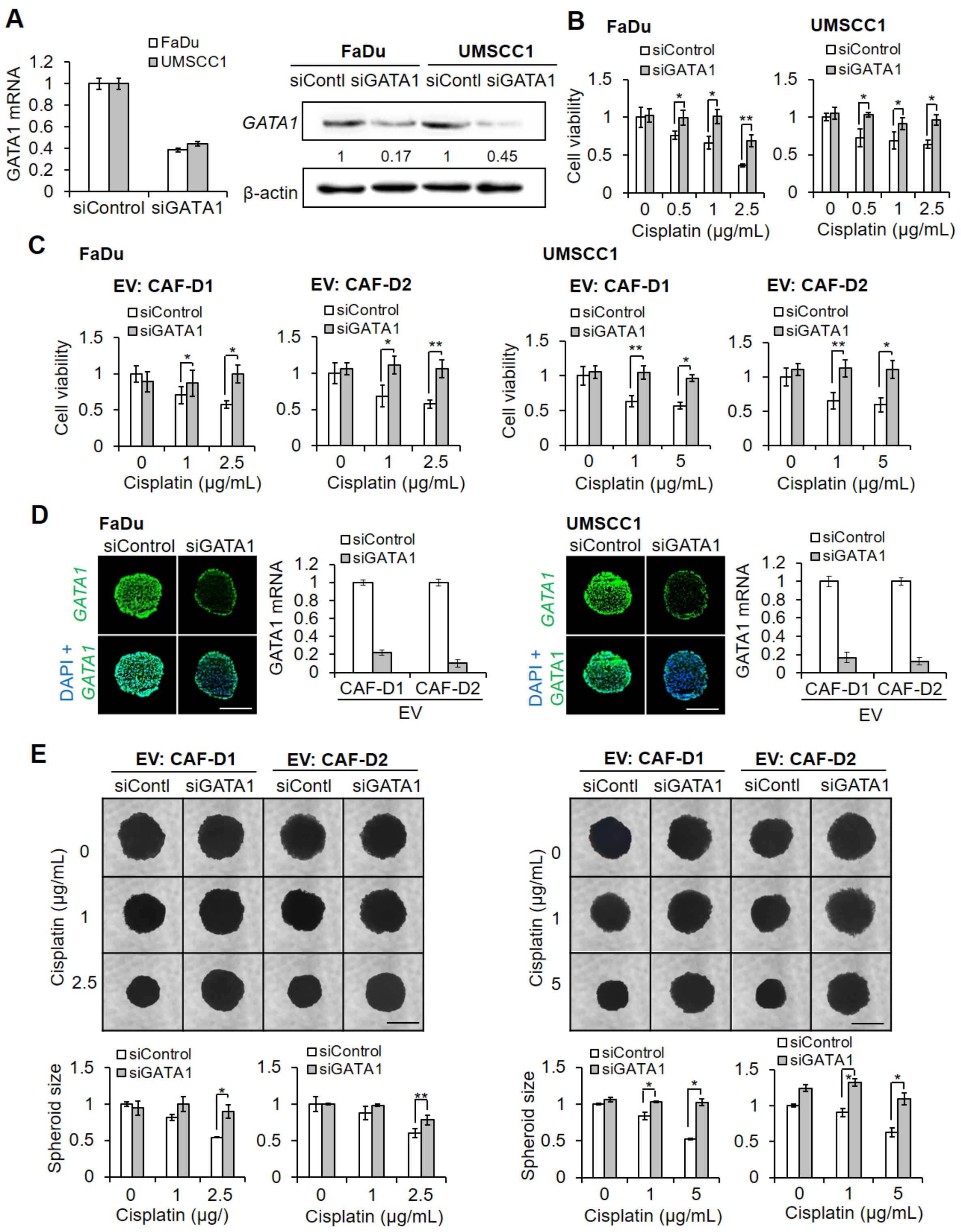
A
FaDu
UMSCC1
GATA1 mRNA
siControl
siGATA1
siContl
GATA1
1
0.17
1
0.45
β-actin
B
Cell viability
Cisplatin (μg/mL)
C
EV: CAF-D1
EV: CAF-D2
D
DAPI + GATA1
CAF-D1
CAF-D2
EV
E
Spheroid size
Cisplatin (μg/)

Figure S6. Effect of *siGATA1* on cisplatin sensitivity in cells or spheroids under CAF-D EV treatment. (A) OSCC cells were transfected with *siGATA1* for 24 h, followed by cisplatin treatment for another 24 h. The mRNA and protein expression of *GATA1* was analyzed. **(B)** Cell viability was evaluated using MTT assay under the same condition. **(C)** OSCC cells were transfected with *siGATA1* for 24 h, followed by CAF-D EV treatment (MOI = 100) for 16 h. After cisplatin treatment for another 24 h, cell viability was evaluated using MTT assay. **(D)** FaDu and UMSCC1 spheroids were formed via culture in 96-well U-bottom ultra-low attachment plates for two days. After transfection with *siGATA1* for 24 h, CAF-D EV treatment was carried out for 16 h. After cisplatin treatment for another 14 days, mRNA and protein expression was analyzed. **(E)** Spheroids were imaged using phase-contrast microscopy, and their sizes were measured using Cell[3]iMager. Each experimental group consisted of eight spheroids, and a representative image is presented. Results were presented as the mean ± standard deviation of three experiments. *$p < 0.05$; **$p < 0.01$. Scale bars: **D-E** 500 μm.

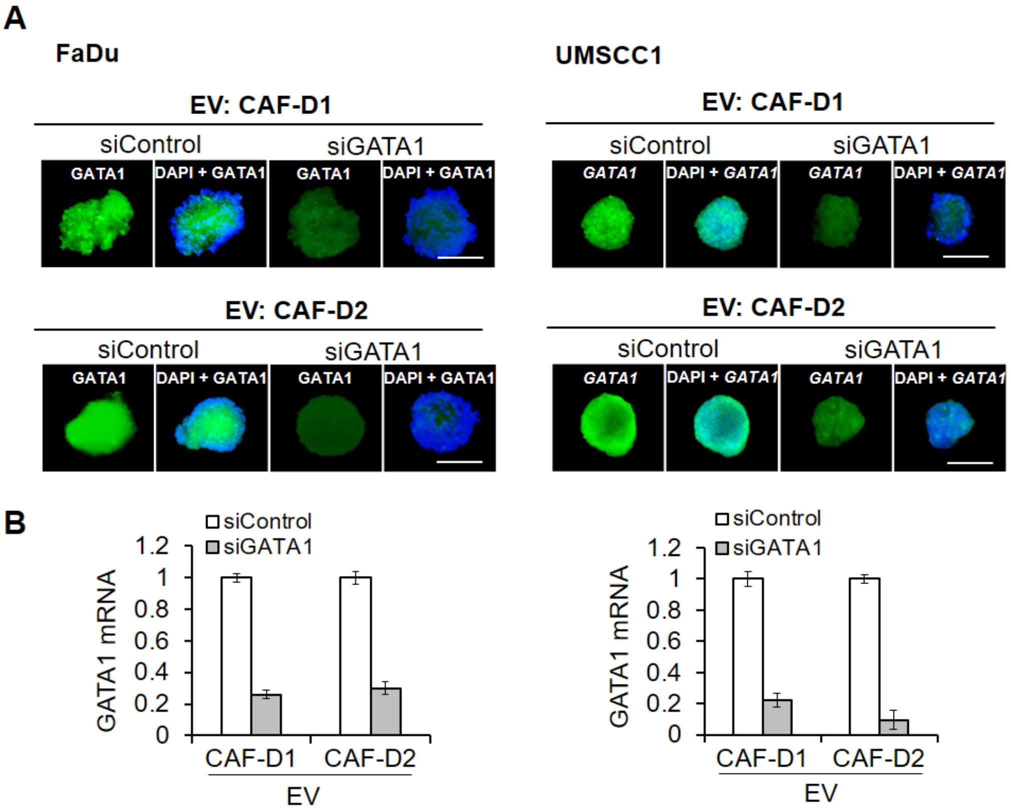

Figure S7. siRNA-dependent reduction of GATA1 mRNA and protein expression in organoids. Organoids derived from OSCC xenografts were transfected with *siGATA1* for 24 h, followed by CAF-D EV treatment (MOI = 100) for another 16 h. **(A)** After cisplatin treatment for seven days, *GATA1* protein expression was compared via IF staining. **(B)** *GATA1* mRNA expression was analyzed using qPCR under the same condition. Results are presented as the mean ± standard deviation of three experiments. Scale bars: **A** 100 μm.

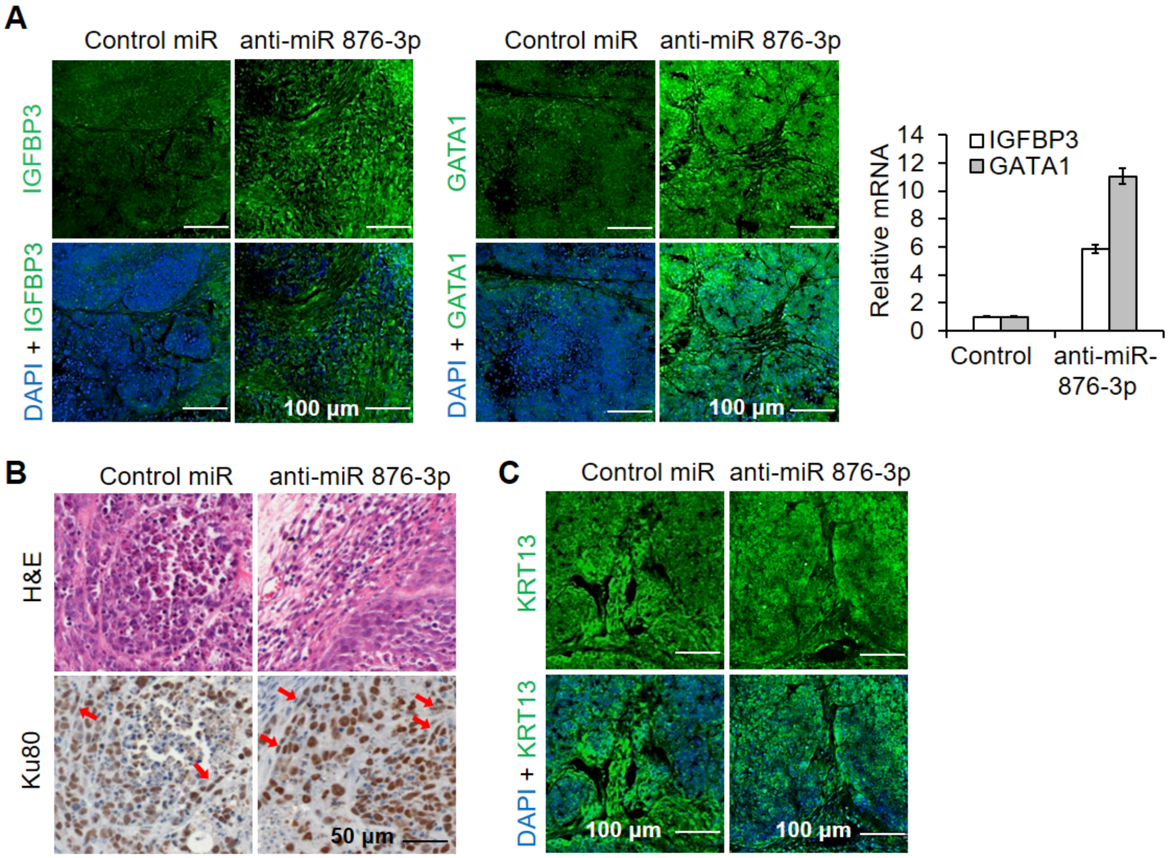

Figure S8. Xenograft characterization and IF staining of *GATA1* and *IGFBP3*. (A) mRNA and protein expression of *IGFBP3* and *GATA1* in xenografts were analyzed using qPCR and IF staining, respectively. **(B)** IHC analysis of mouse tumor tissues was performed with an antibody against Ku80 to evaluate whether these are derived from human cells. Tissues were counterstained with H&E. **(C)** IF staining with the anti-KRT13 antibody revealed squamous epithelial xenograft characteristics.

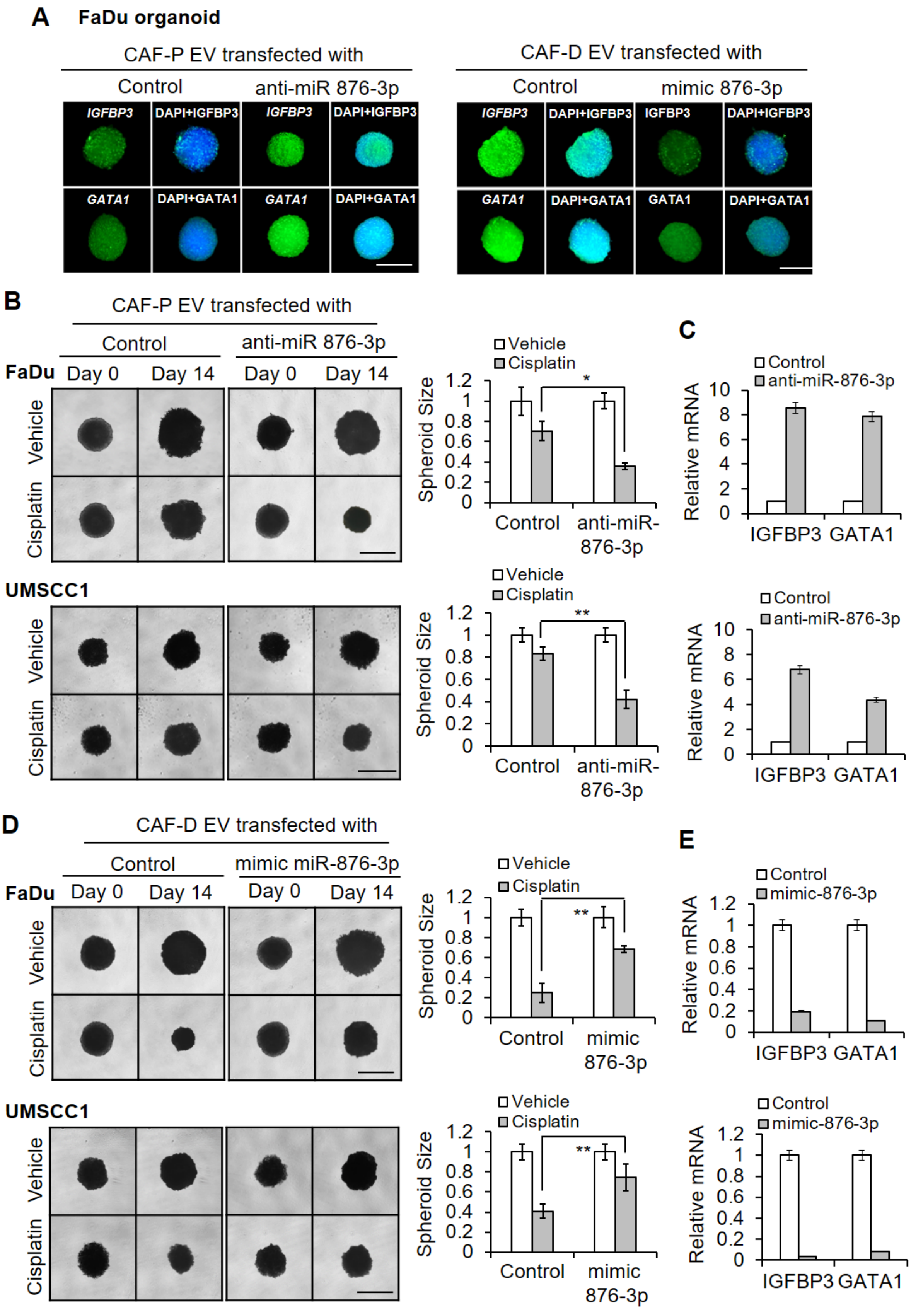

A FaDu organoid
CAF-P EV transfected with
Control
anti-miR 876-3p
IGFBP3
DAPI+IGFBP3
GATA1
DAPI+GATA1
CAF-D EV transfected with
Control
mimic 876-3p
B
CAF-P EV transfected with
Control
anti-miR 876-3p
FaDu
Day 0
Day 14
Vehicle
Cisplatin
Spheroid Size
*
Control
anti-miR-876-3p
UMSCC1
**
C
Control
anti-miR-876-3p
Relative mRNA
IGFBP3
GATA1
D
CAF-D EV transfected with
Control
mimic miR-876-3p
mimic 876-3p
E
Control
mimic-876-3p

Figure S9. Effect of EVs loaded with anti-miR-876-3p or mimic miR-876-3p on cisplatin sensitivity in OSCC. (A) FaDu organoids were incubated with CAF-P EV (MOI = 100) carrying anti-miR-876-3p or CAF-D EV (MOI = 100) carrying mimic miR-876-3p for 16 h, followed by cisplatin treatment for another seven days. *IGFBP3* and *GATA1* protein levels were compared via IF staining. **(B, D)** CAF-P EV carrying anti-miR-876-3p or CAF-D EV carrying mimic miR-876-3p were transfected in FaDu and UMSCC1 spheroids for 16 h, followed by cisplatin treatment. After 14 days, spheroids size was analyzed using Cell^{3}iMager. **(C, E)** mRNA expression of *IGFBP3* and *GATA1* was analyzed in spheroids on day 14. Results are presented as the mean ± standard deviation from three experiments. *$p < 0.05$; **$p < 0.01$. Scale bars: **A, B, D** 500 μm.
